# Supplementary material for: Computed tomography-based radiomic features combined with clinical parameters for predicting post-infectious bronchiolitis obliterans in children with adenovirus pneumonia: a retrospective study
Source: PeerJ. 2025 Mar 31;13:e19145. doi: 10.7717/peerj.19145 (PMC11967419; doi:10.7717/peerj.19145)
Supplement: Supplemental Information 1 [file peerj-13-19145-s001.docx]

| Features Represent | Filter | Type | Features | Coefficients |
| --- | --- | --- | --- | --- |
| Feature 1 | original | shape | maximum 2d diameter column | 0.072 |
| Feature 2 | box sigma image | glszm | zone entropy | 0.061 |
| Feature 3 | speckle noise | glcm | imc2 | 0.059 |
| Feature 4 | wavelet-hhl | glcm | idmn | 0.055 |
| Feature 5 | wavelet-lhh | first-order | median | 0.028 |
| Feature 6 | wavelet-hhl | glszm | zone entropy | 0.004 |
| Feature 7 | box sigma image | gldm | dependence non uniformity | 0.003 |
| Feature 8 | additive gaussian noise | first-order | root mean squared | -0.037 |
| Feature 9 | wavelet-hlh | glcm | cluster shade | -0.055 |
| Feature 10 | wavelet-hlh | glszm | size zone non uniformity normalized | -0.086 |
