# Supplementary material for: Computed tomography-based radiomic features combined with clinical parameters for predicting post-infectious bronchiolitis obliterans in children with adenovirus pneumonia: a retrospective study
Source: PeerJ. 2025 Mar 31;13:e19145. doi: 10.7717/peerj.19145 (PMC11967419; doi:10.7717/peerj.19145)
Supplement: Supplemental Information 3 [file peerj-13-19145-s003.zip › Raw date/Figure 1.pptx]

## Slide 1
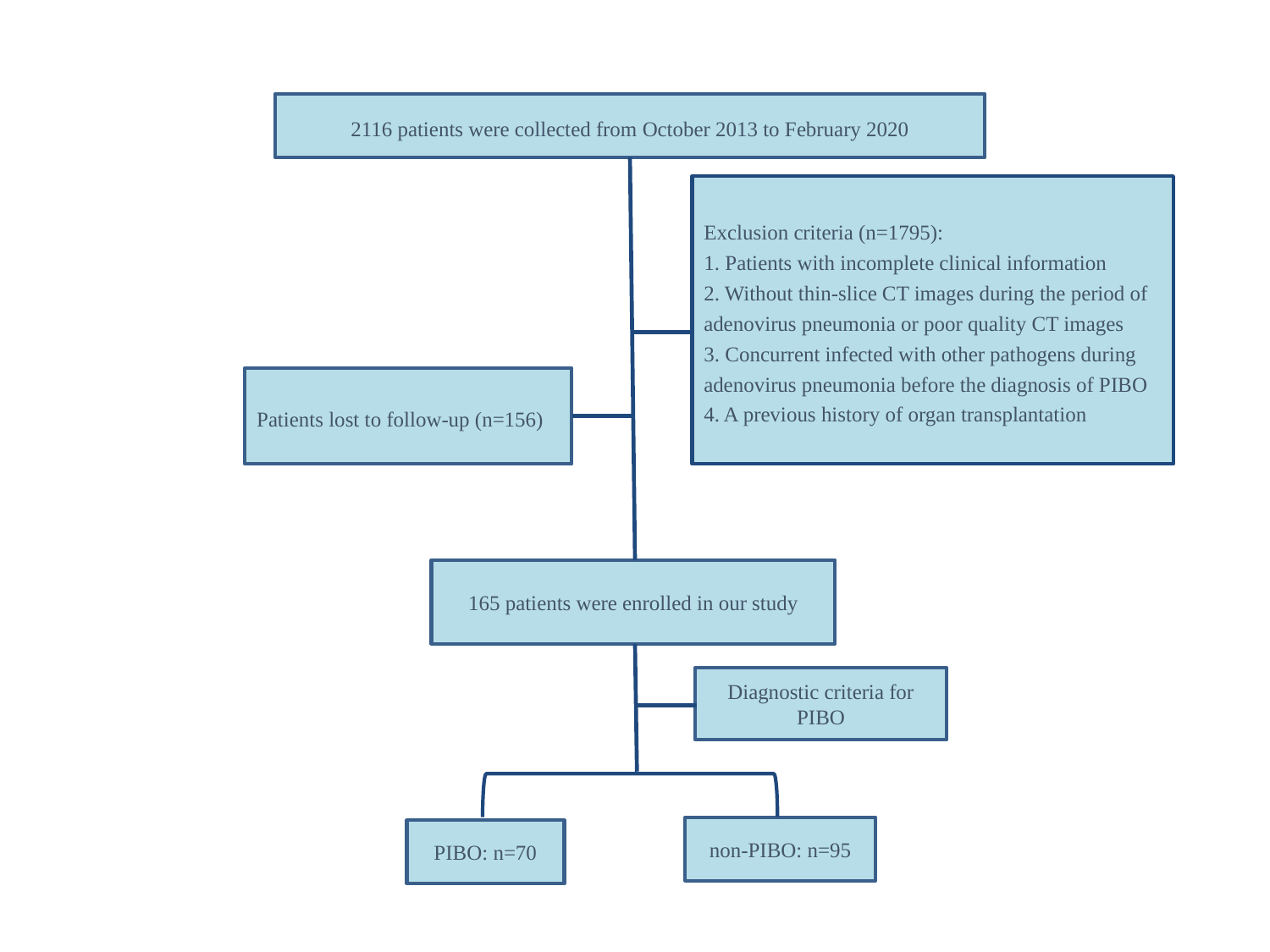

2116 patients were collected from October 2013 to February 2020
Exclusion criteria (n=1795):
1. Patients with incomplete clinical information
2. Without thin-slice CT images during the period of adenovirus pneumonia or poor quality CT images
3. Concurrent infected with other pathogens during adenovirus pneumonia before the diagnosis of PIBO
4. A previous history of organ transplantation
Patients lost to follow-up (n=156)
165 patients were enrolled in our study
Diagnostic criteria for PIBO
non-PIBO: n=95
PIBO: n=70
