# Supplementary figures and images for: Computed tomography-based radiomic features combined with clinical parameters for predicting post-infectious bronchiolitis obliterans in children with adenovirus pneumonia: a retrospective study
Source: PeerJ. 2025 Mar 31;13:e19145. doi: 10.7717/peerj.19145 (PMC11967419; doi:10.7717/peerj.19145)

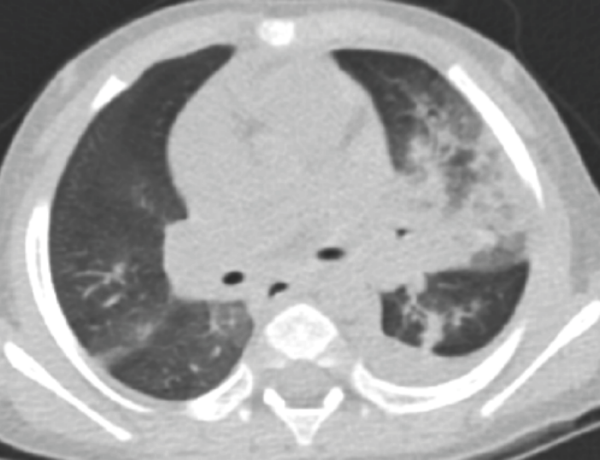

Supplement: Supplemental Information 3 [file peerj-13-19145-s003.zip › Raw date/Figure 2/Figure 2-a.png]

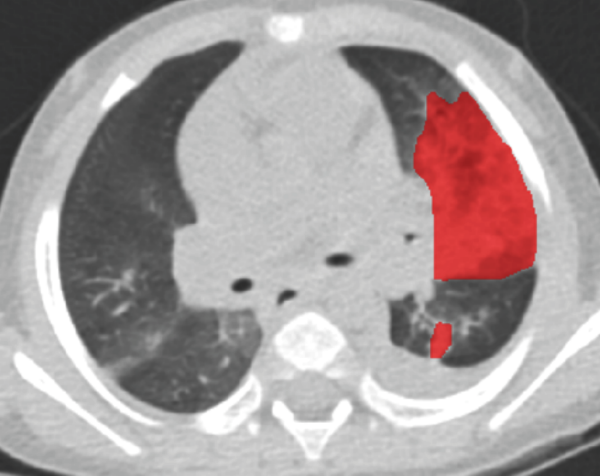

Supplement: Supplemental Information 3 [file peerj-13-19145-s003.zip › Raw date/Figure 2/Figure 2-b.png]

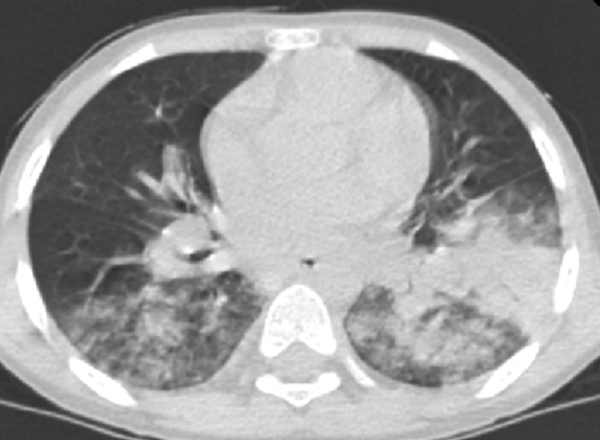

Supplement: Supplemental Information 3 [file peerj-13-19145-s003.zip › Raw date/Figure 2/Figure 2-c.png]

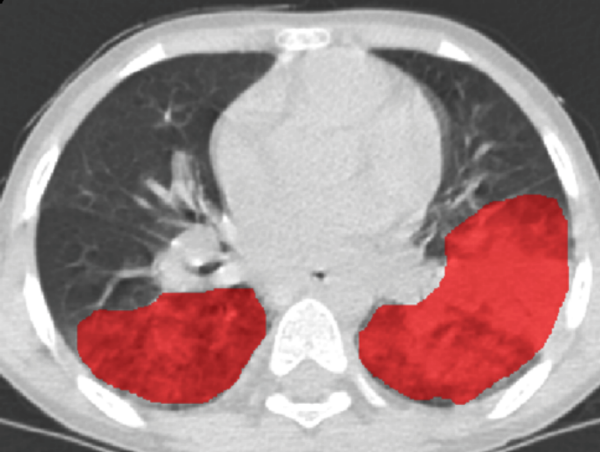

Supplement: Supplemental Information 3 [file peerj-13-19145-s003.zip › Raw date/Figure 2/Figure 2-d.png]

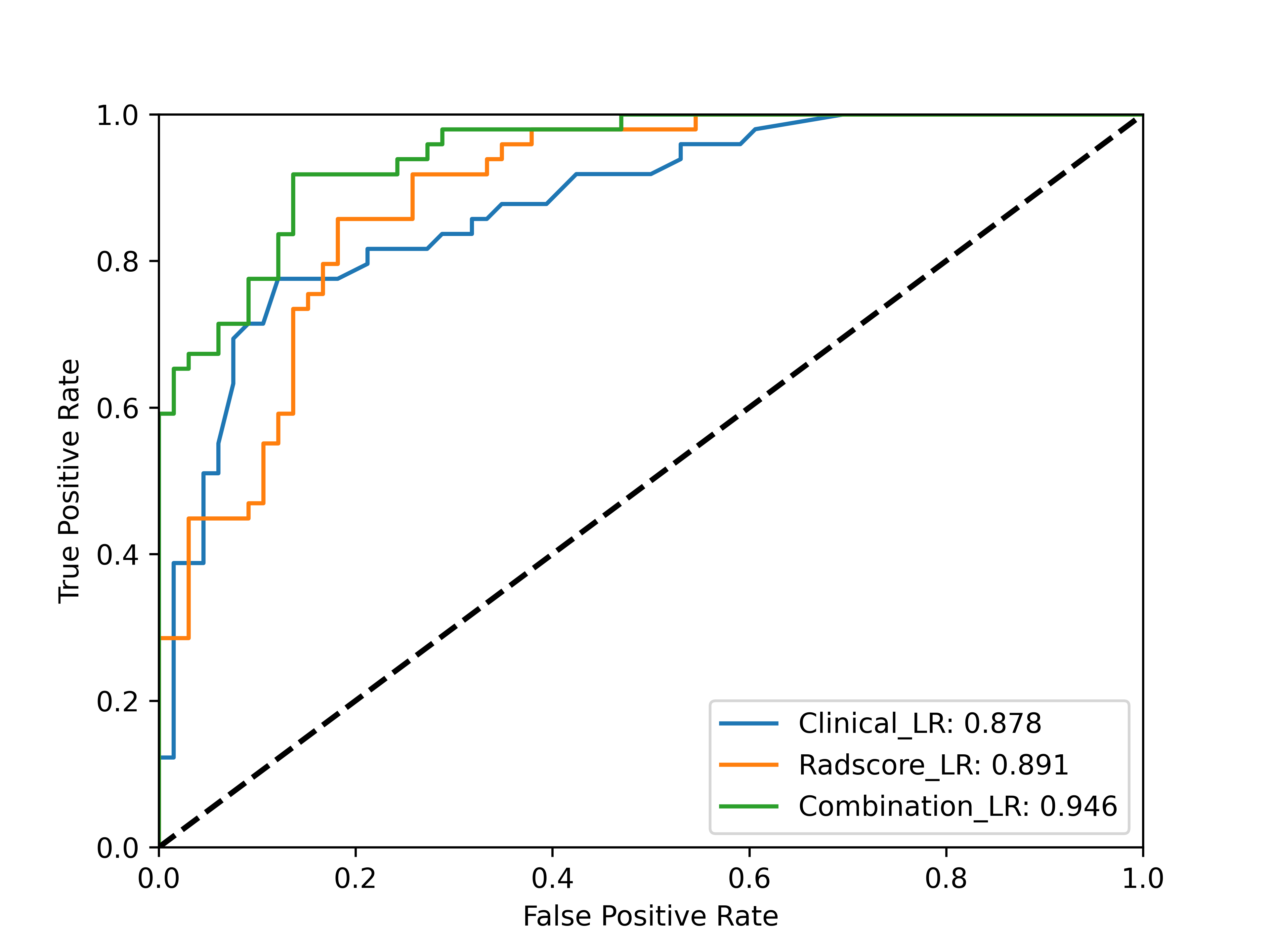

Supplement: Supplemental Information 3 [file peerj-13-19145-s003.zip › Raw date/Figure 3/3-a.tiff]

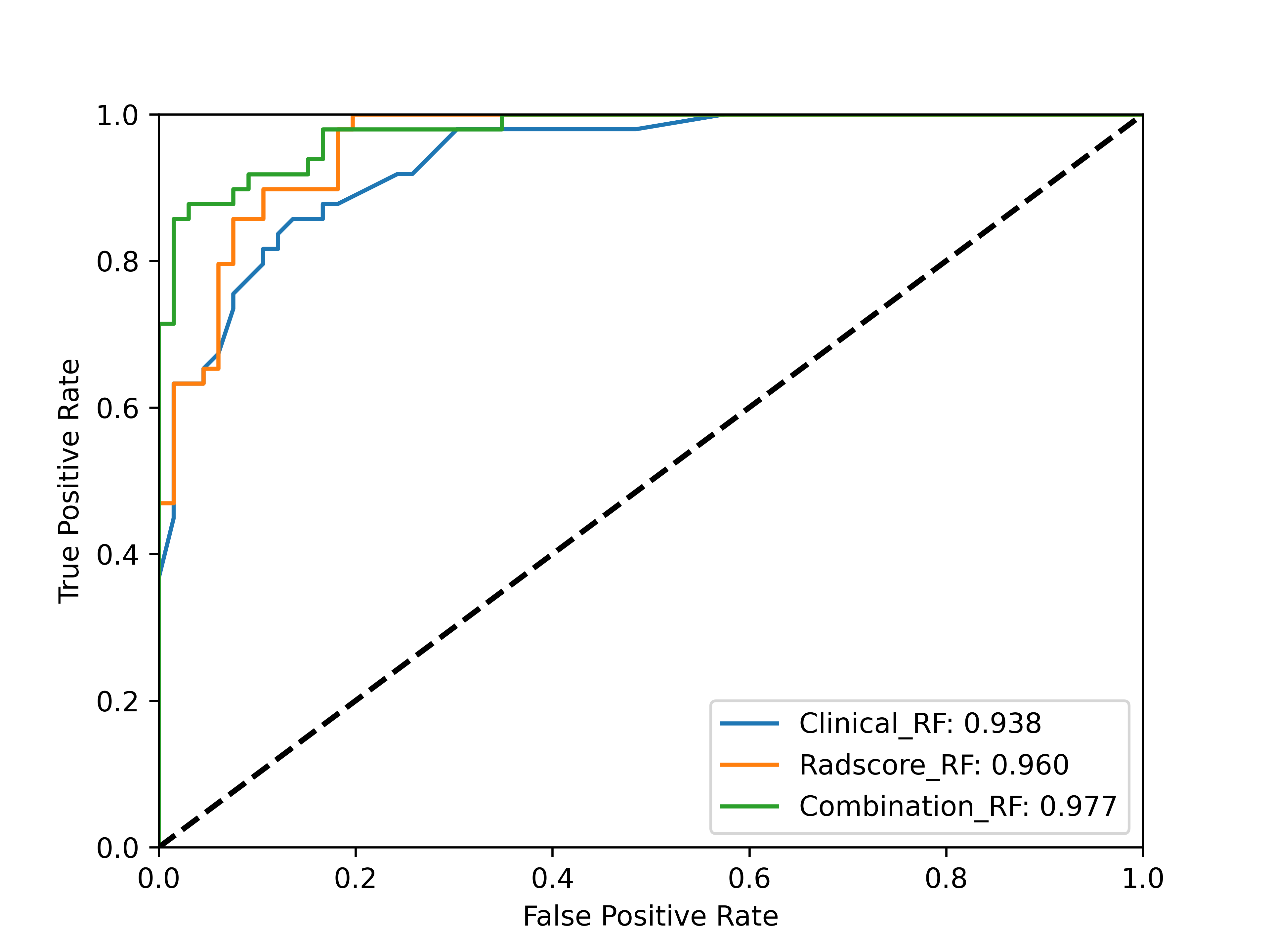

Supplement: Supplemental Information 3 [file peerj-13-19145-s003.zip › Raw date/Figure 3/3-b.tiff]

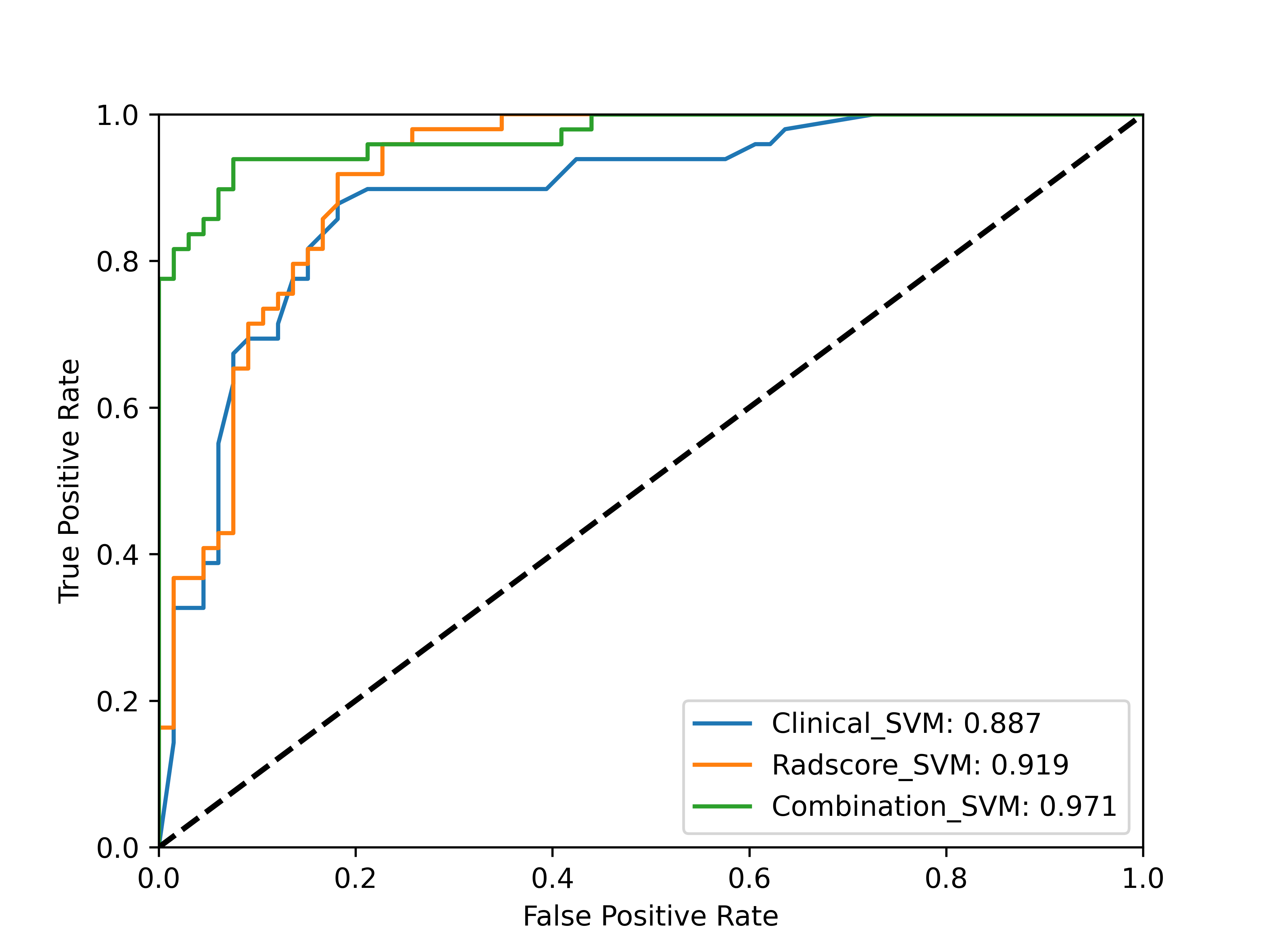

Supplement: Supplemental Information 3 [file peerj-13-19145-s003.zip › Raw date/Figure 3/3-c.tiff]

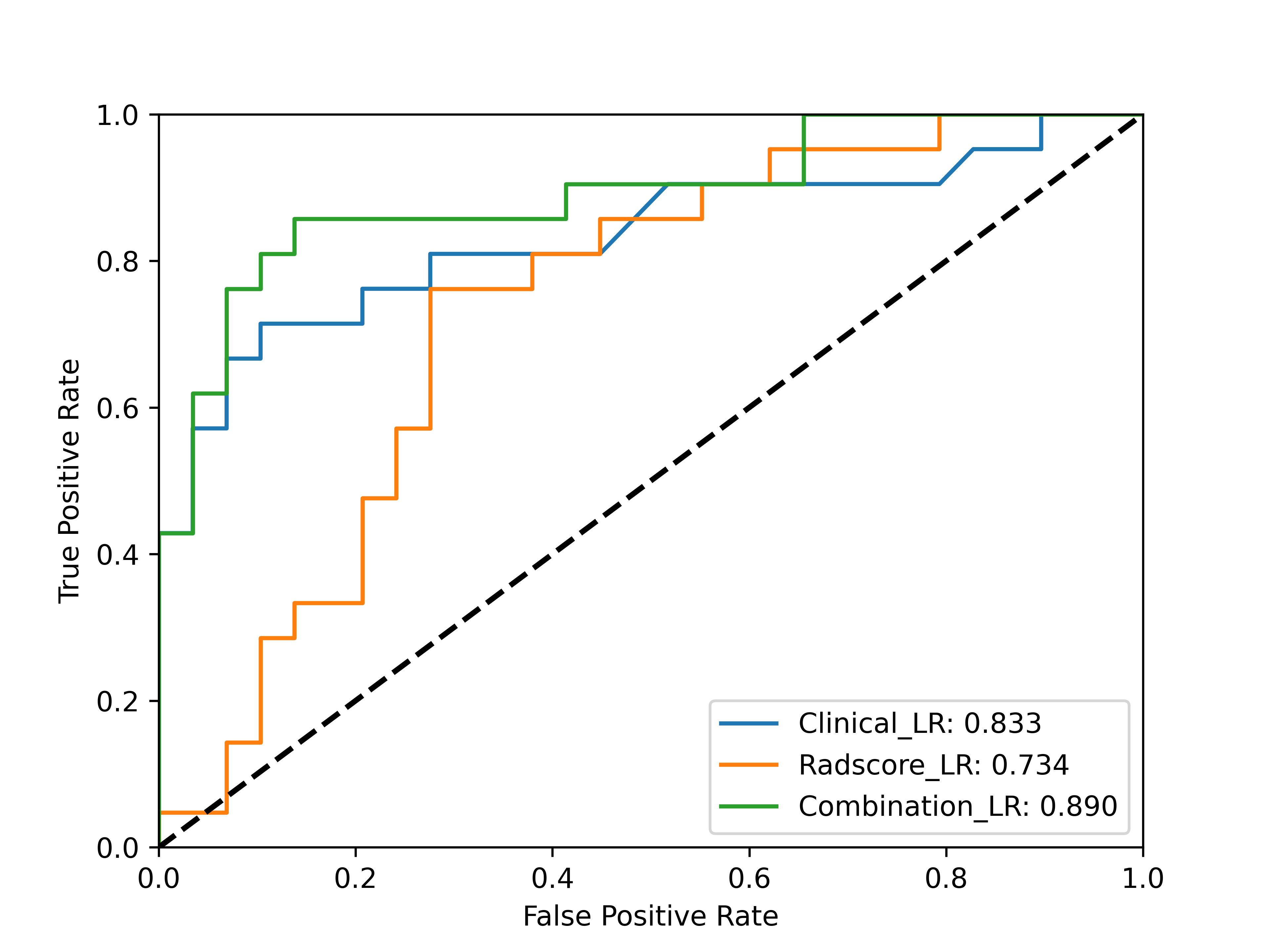

Supplement: Supplemental Information 3 [file peerj-13-19145-s003.zip › Raw date/Figure 3/3-d.tiff]

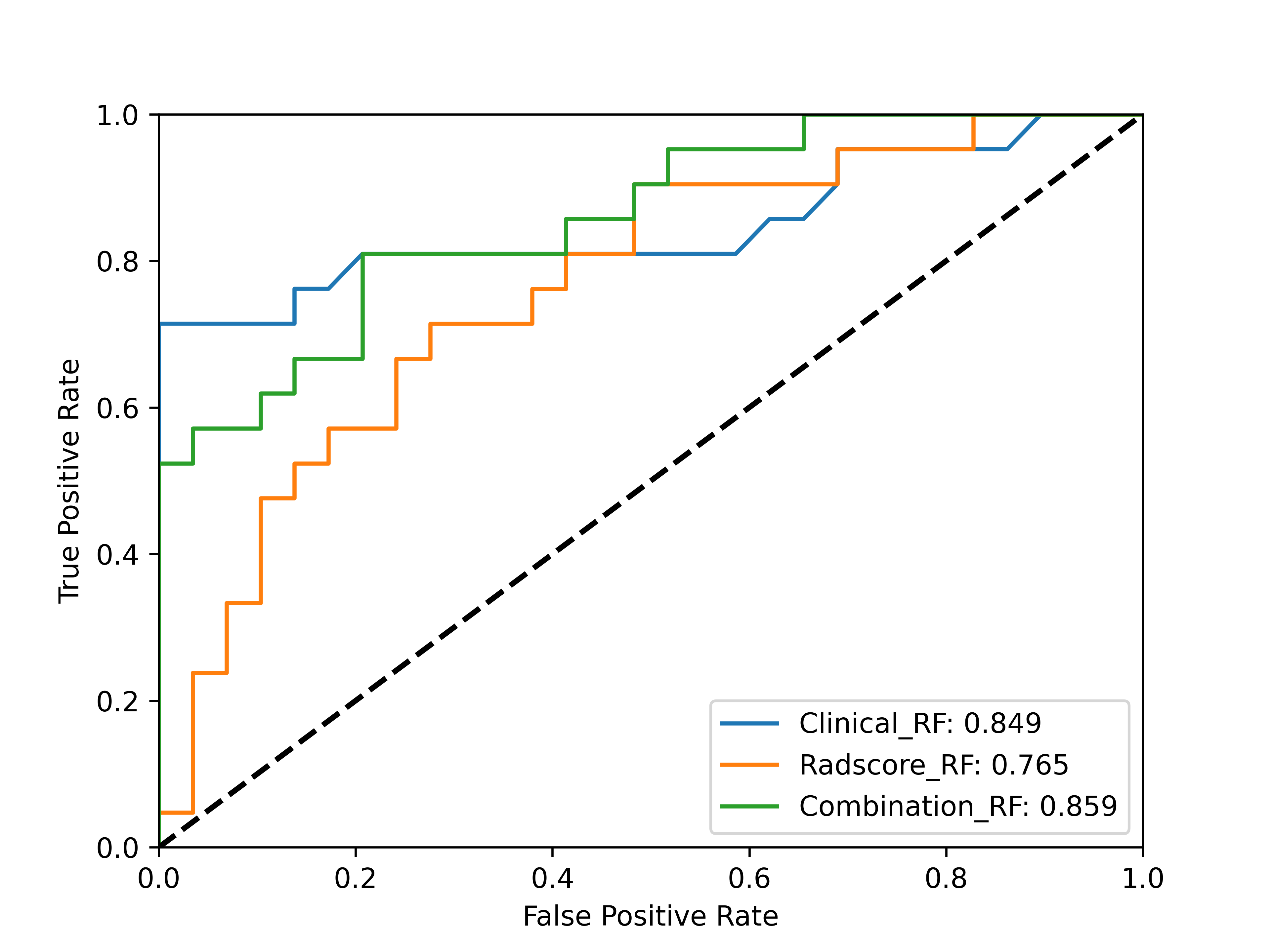

Supplement: Supplemental Information 3 [file peerj-13-19145-s003.zip › Raw date/Figure 3/3-e.tiff]

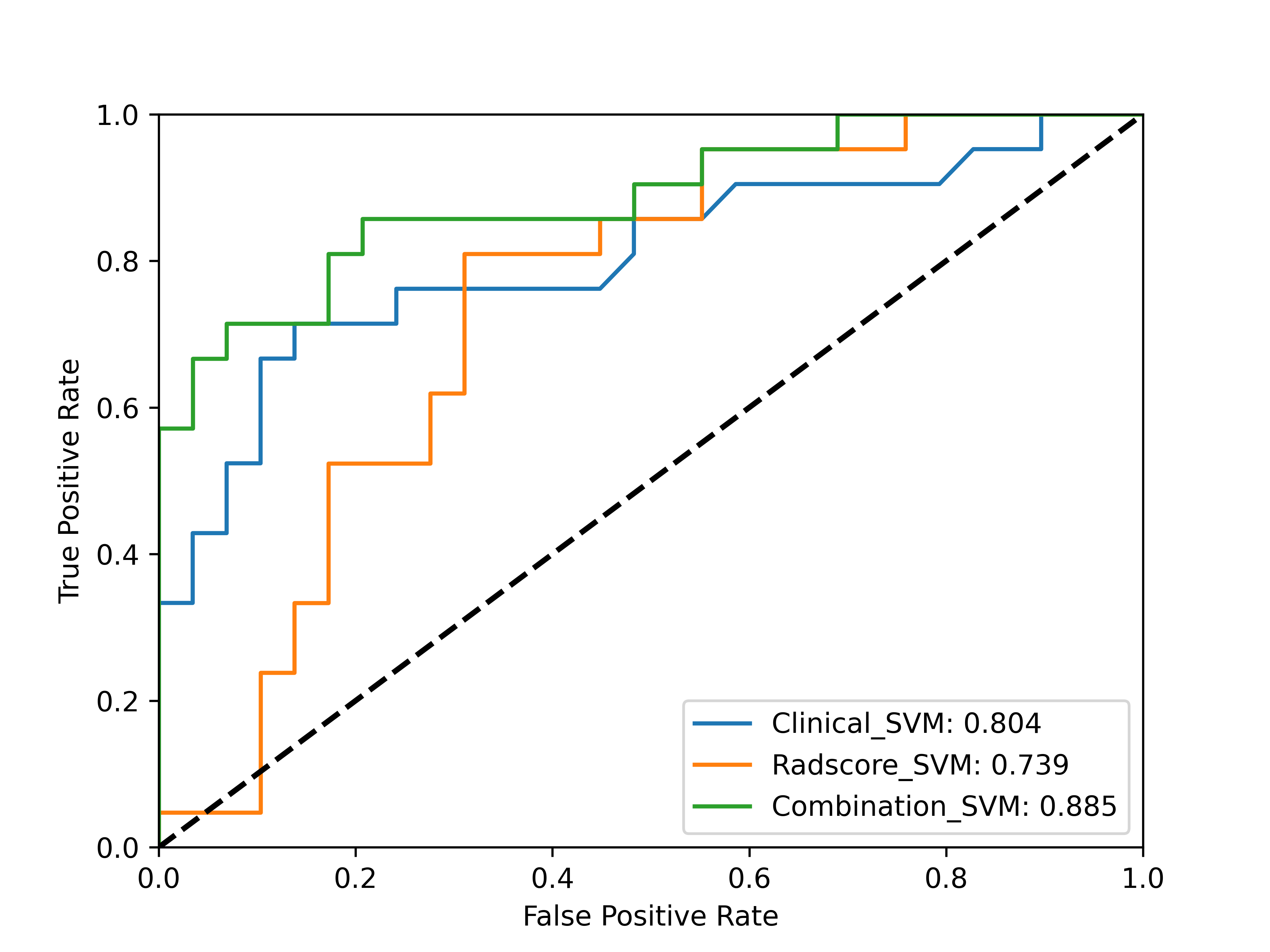

Supplement: Supplemental Information 3 [file peerj-13-19145-s003.zip › Raw date/Figure 3/3-f.tiff]

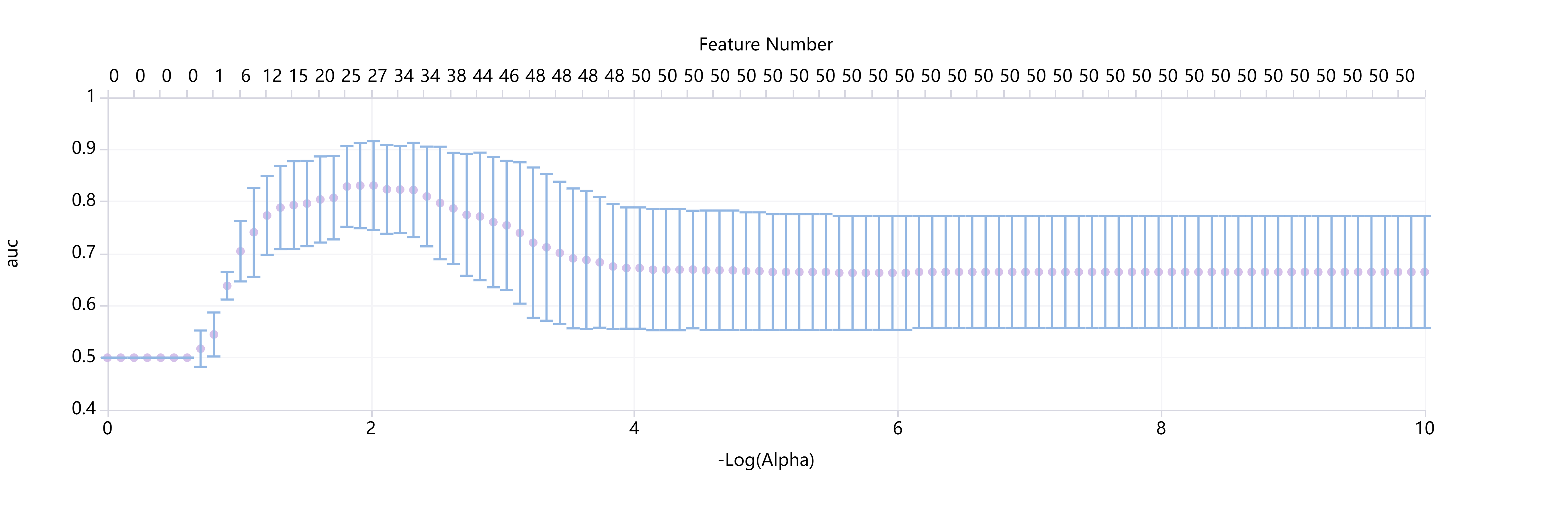

Supplement: Supplemental Information 3 [file peerj-13-19145-s003.zip › Raw date/Figure 4/Figure 4-a.tiff]

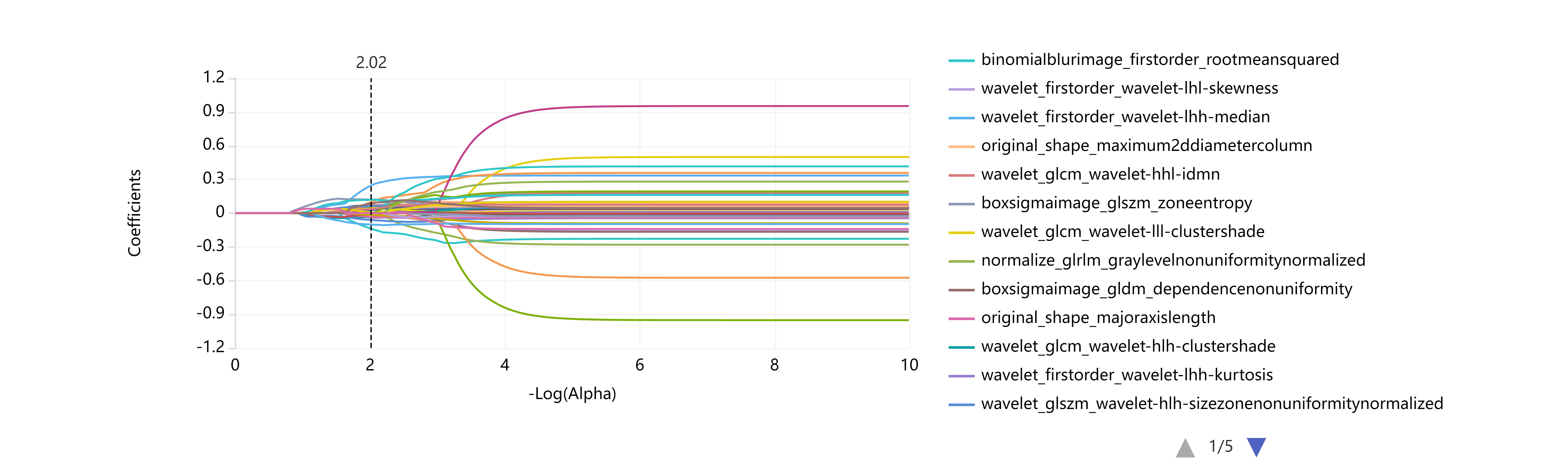

Supplement: Supplemental Information 3 [file peerj-13-19145-s003.zip › Raw date/Figure 4/Figure 4-b.tiff]

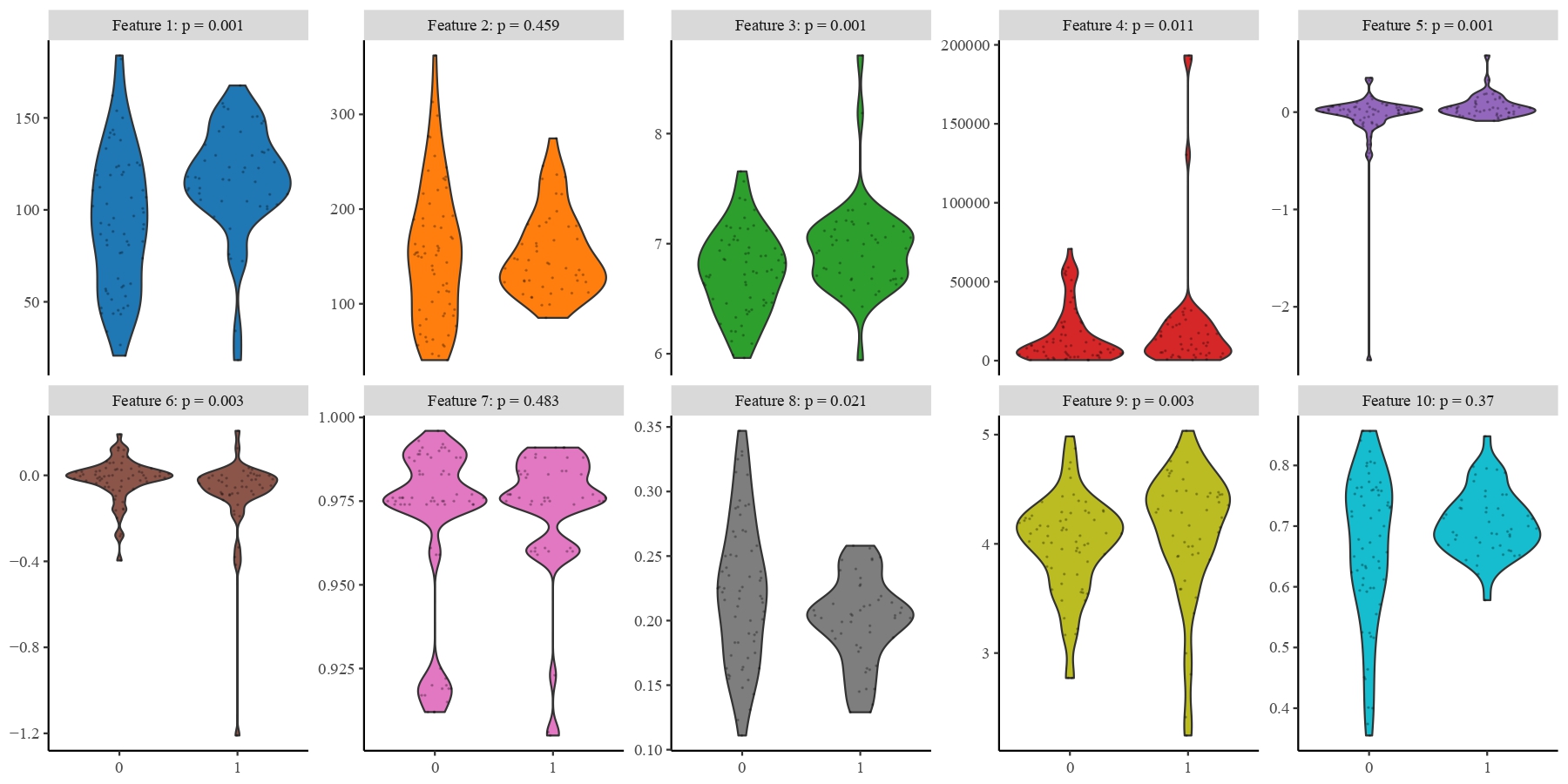

Supplement: Supplemental Information 3 [file peerj-13-19145-s003.zip › Raw date/Figure 4/Figure 4-c.jpg]

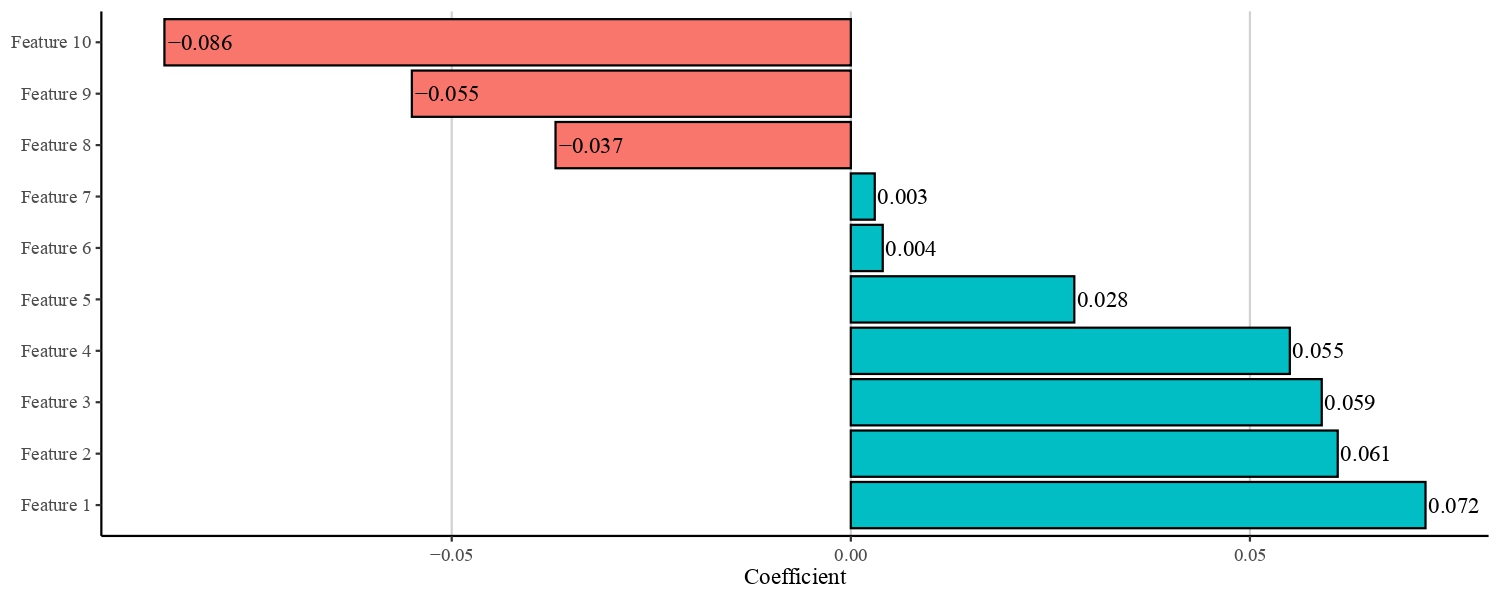

Supplement: Supplemental Information 3 [file peerj-13-19145-s003.zip › Raw date/Figure 4/Figure 4-d.jpg]

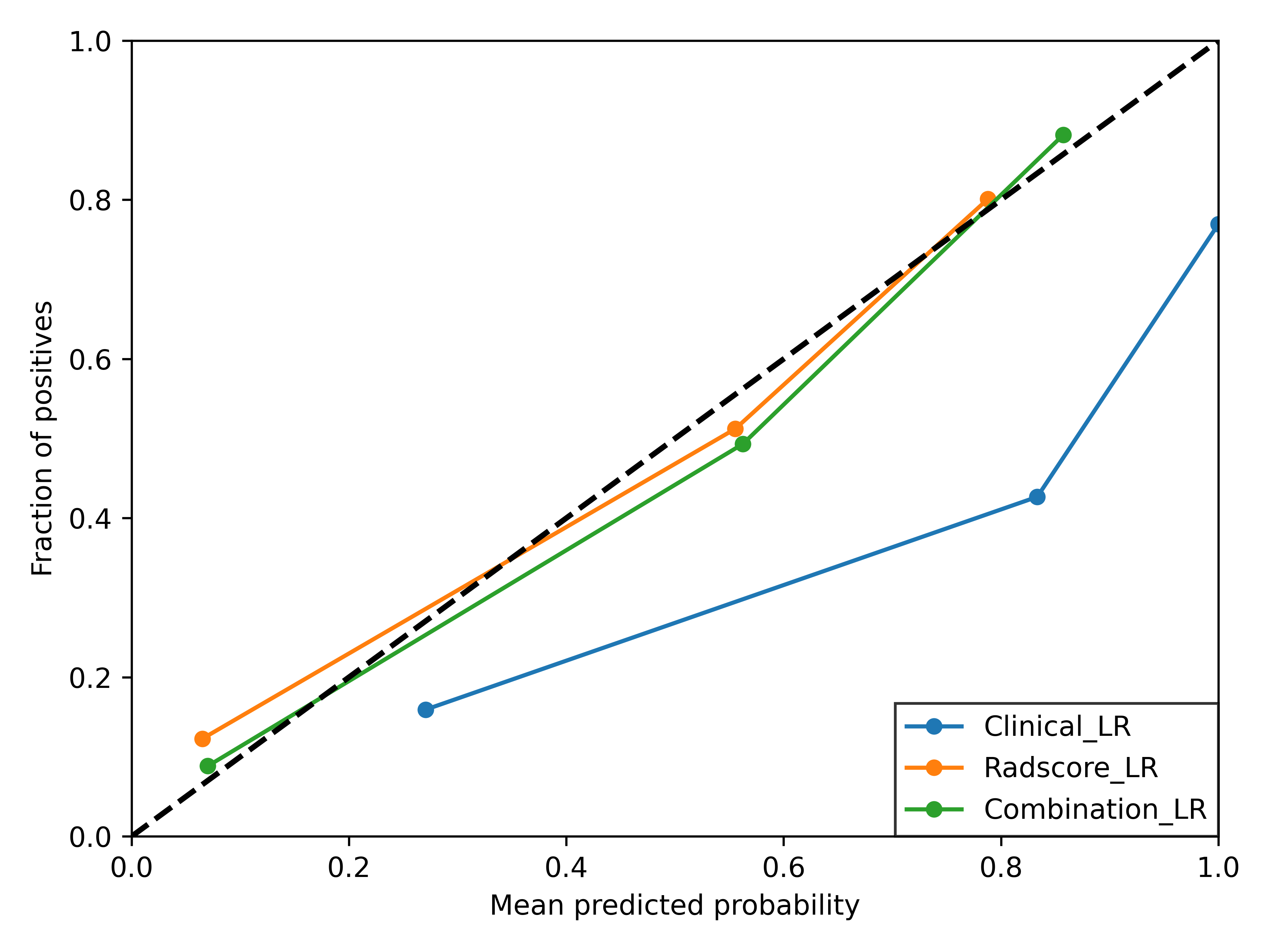

Supplement: Supplemental Information 3 [file peerj-13-19145-s003.zip › Raw date/Figure 5/5-a.tiff]

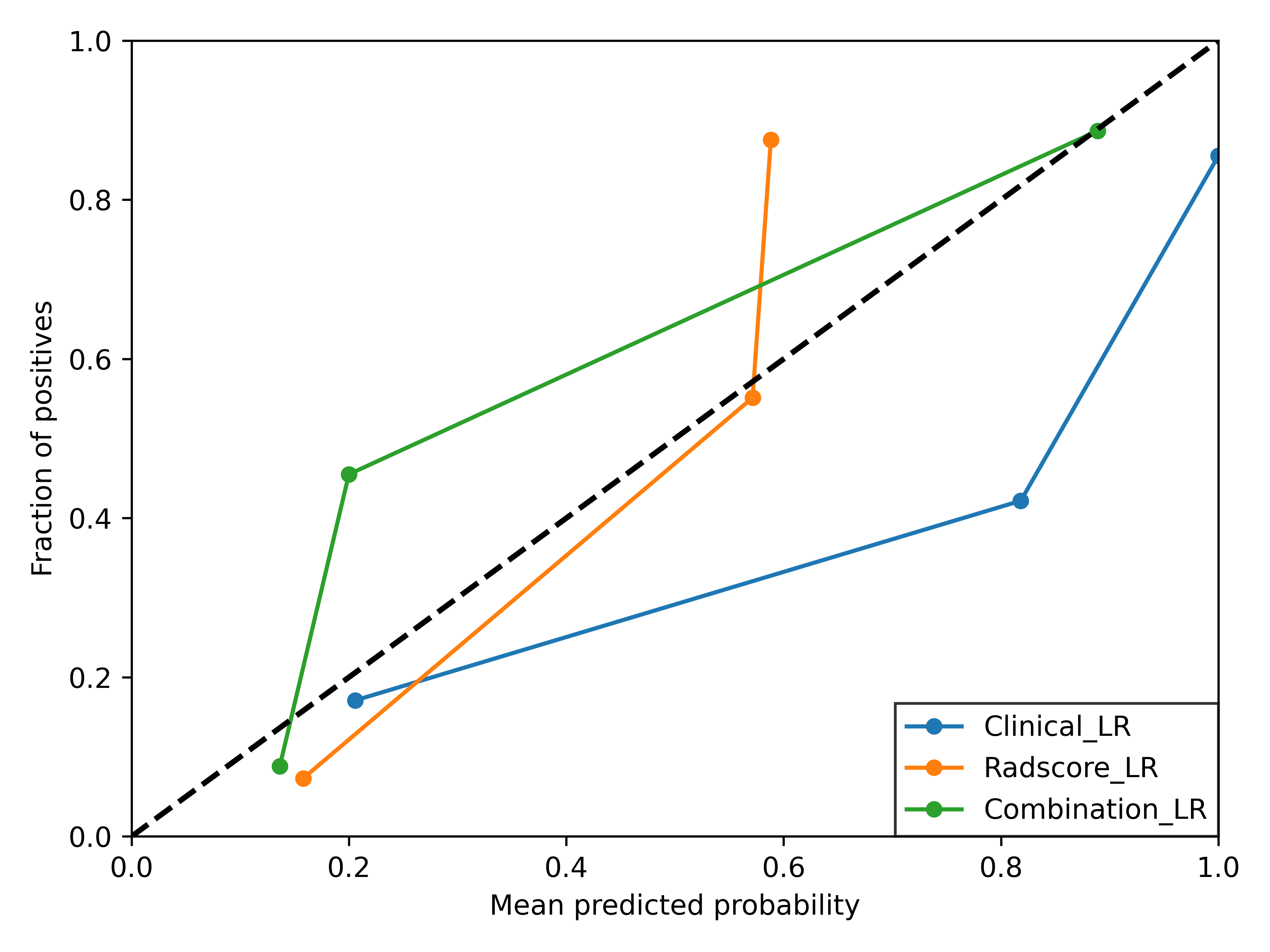

Supplement: Supplemental Information 3 [file peerj-13-19145-s003.zip › Raw date/Figure 5/5-b.tiff]

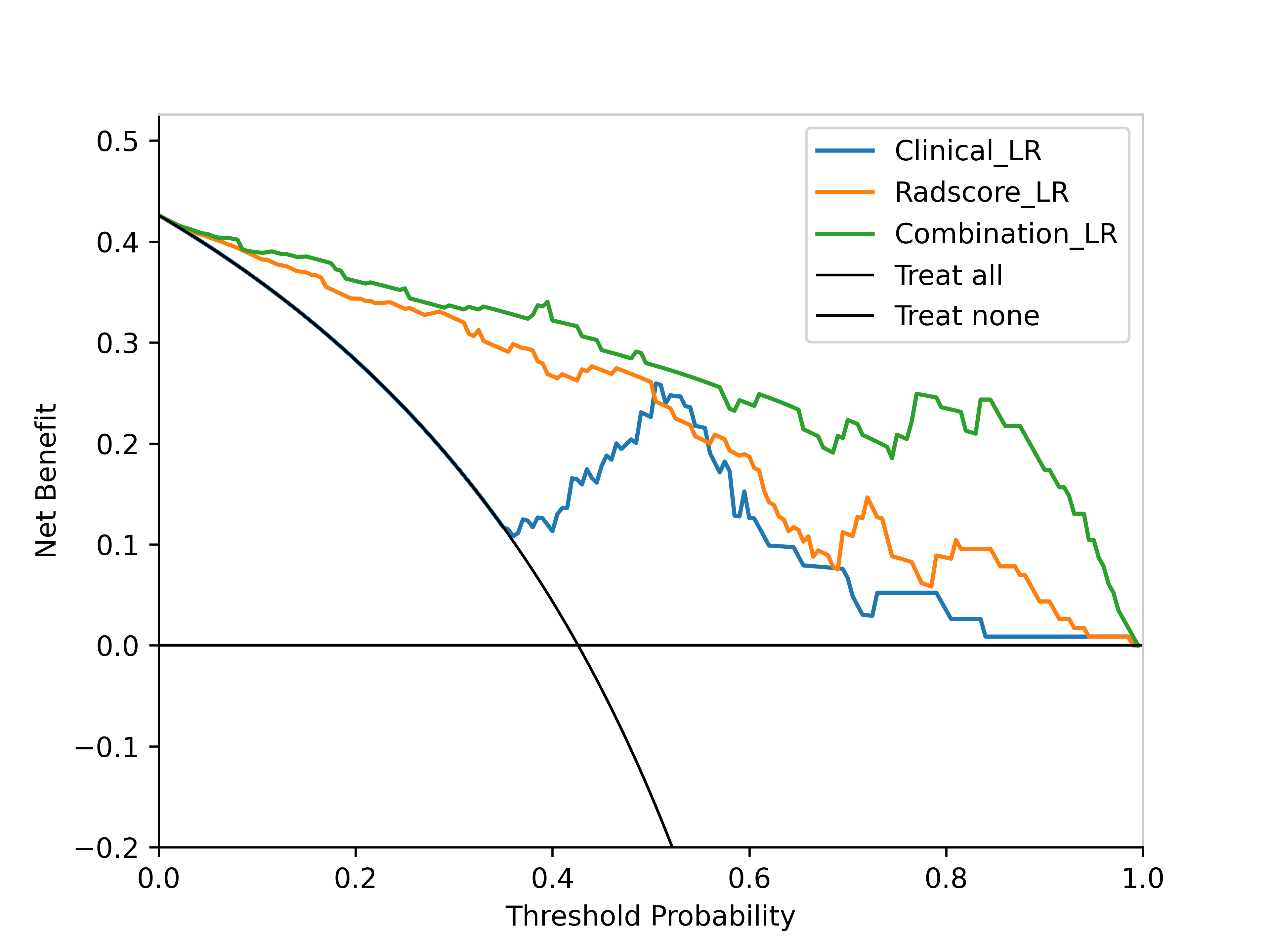

Supplement: Supplemental Information 3 [file peerj-13-19145-s003.zip › Raw date/Figure 5/5-c.tiff]

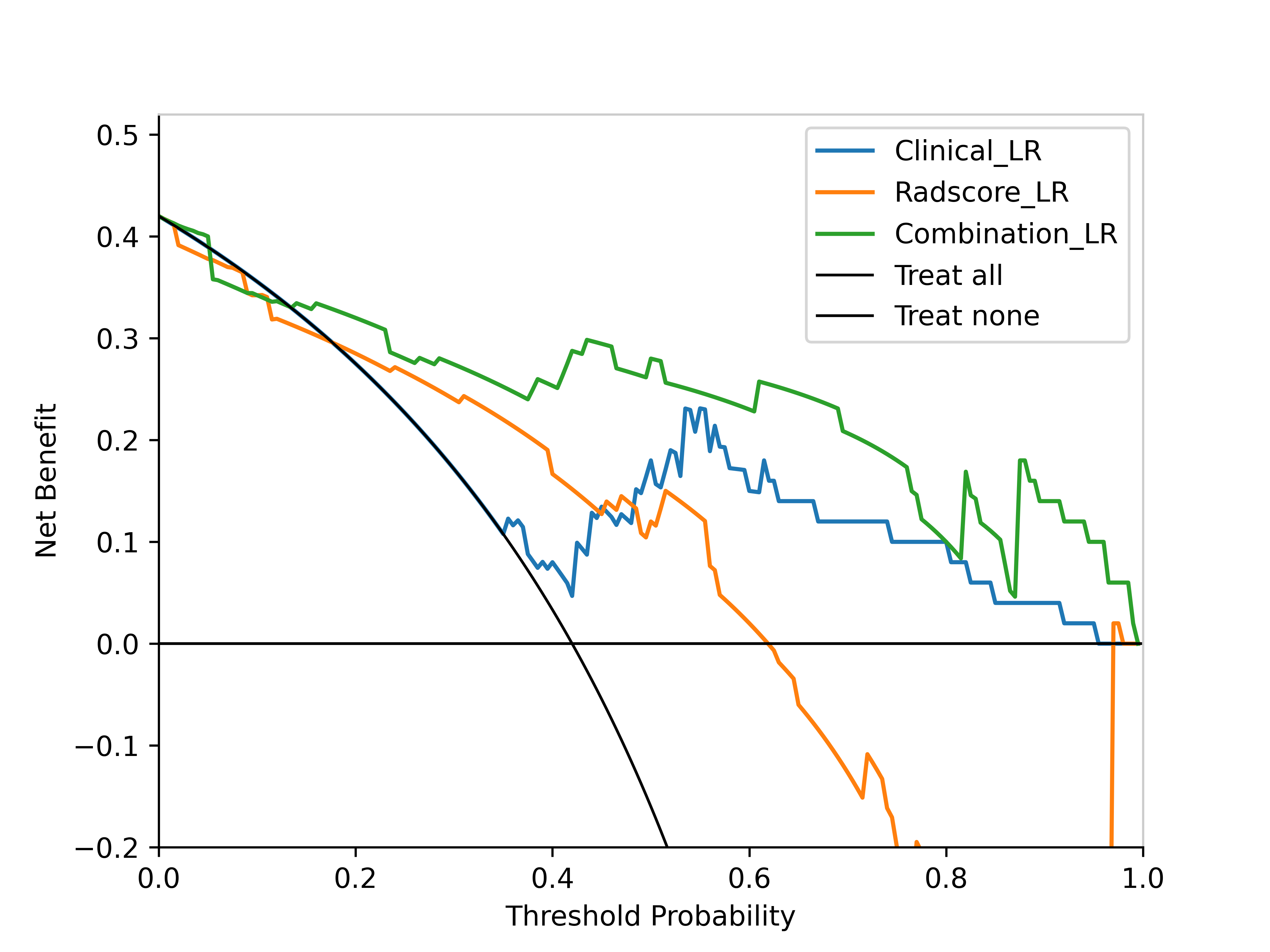

Supplement: Supplemental Information 3 [file peerj-13-19145-s003.zip › Raw date/Figure 5/5-d.tiff]

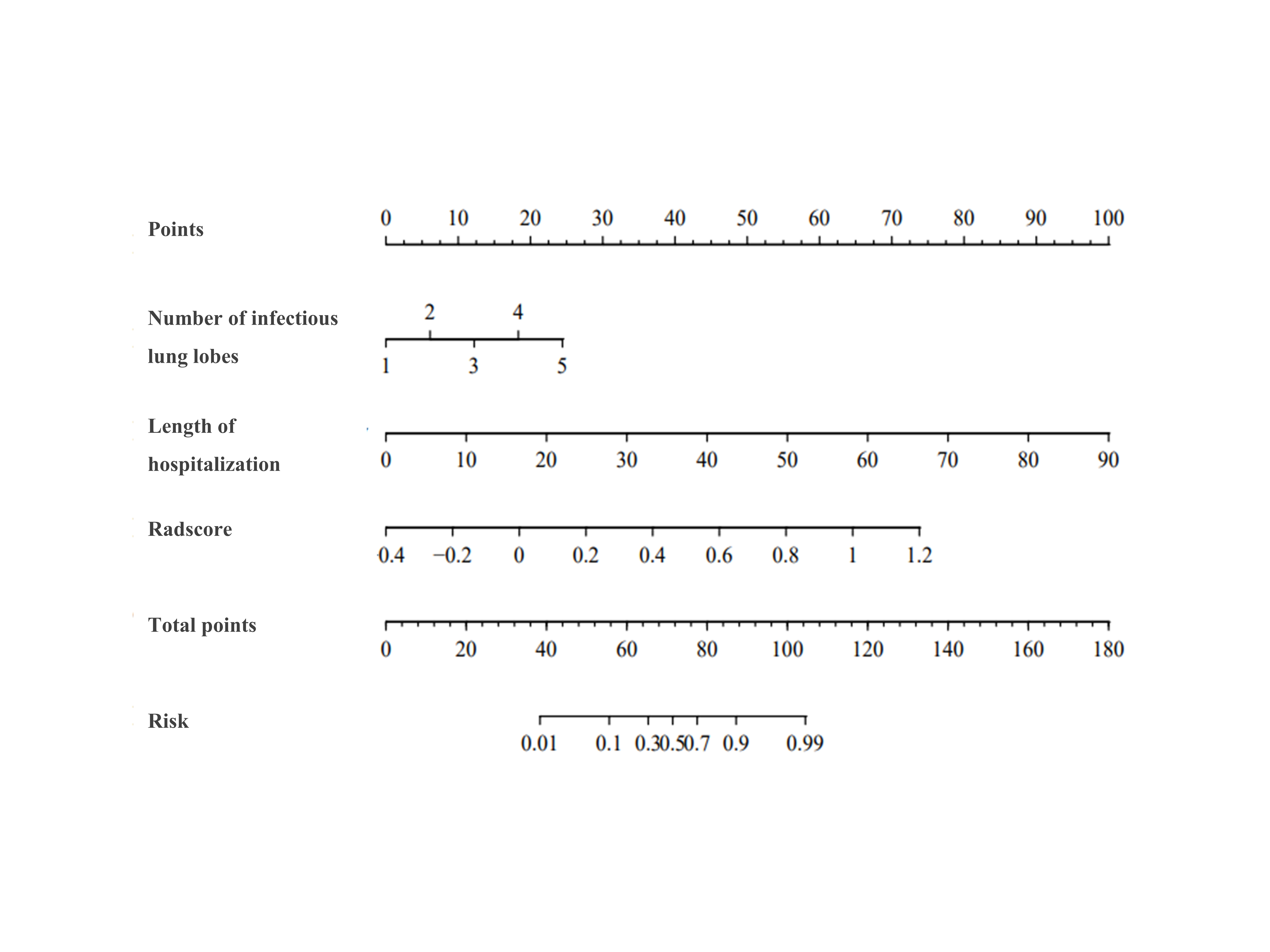

Supplement: Supplemental Information 3 [file peerj-13-19145-s003.zip › Raw date/Figure 6/Fig 6.tif]

## Slide 1
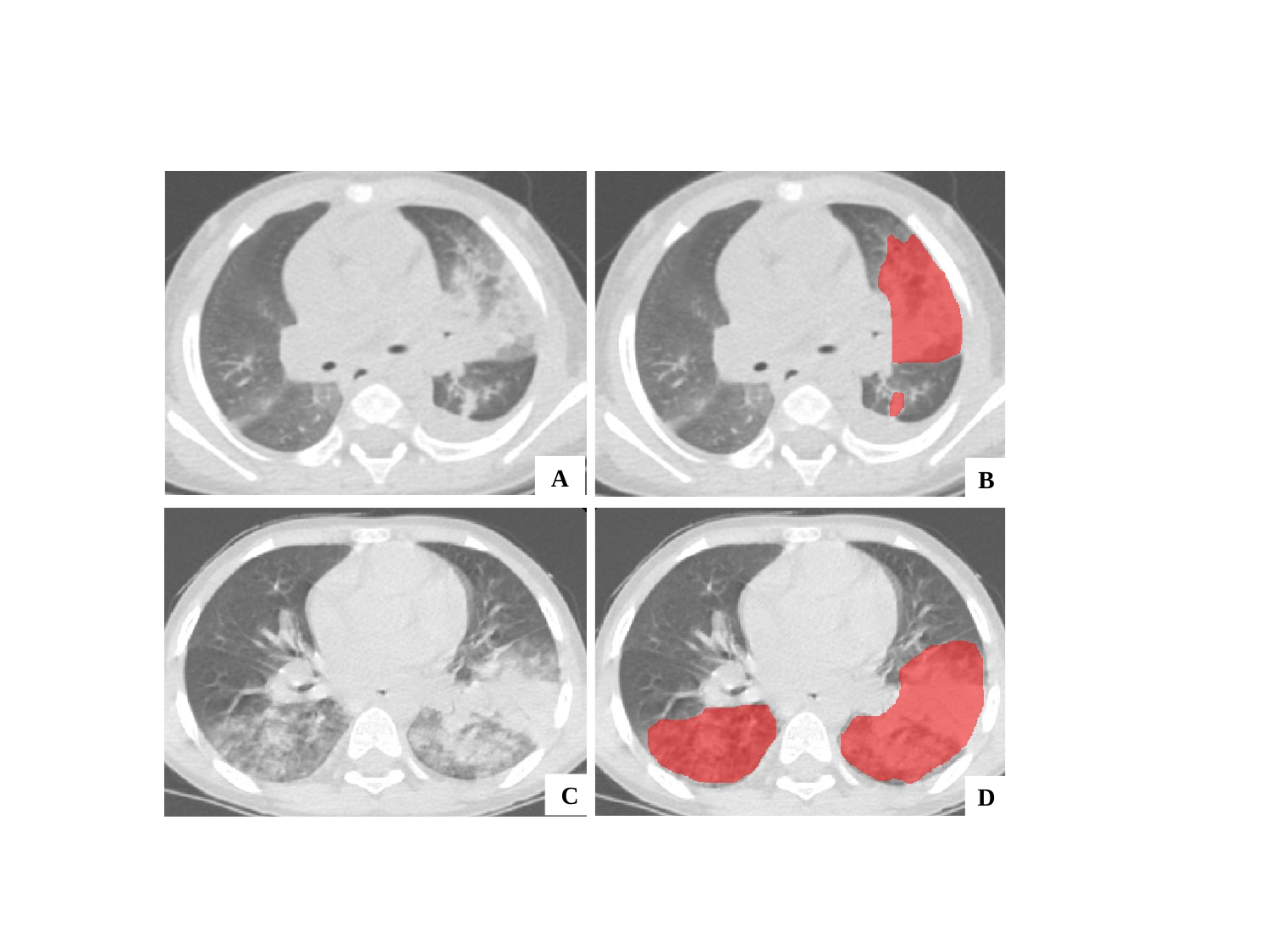

A
B
C
D

Supplement: Supplemental Information 3 [file peerj-13-19145-s003.zip › Raw date/Figure 6/Figure 6.pptx]
