# Supplementary material for: Computed tomography-based radiomic features combined with clinical parameters for predicting post-infectious bronchiolitis obliterans in children with adenovirus pneumonia: a retrospective study
Source: PeerJ. 2025 Mar 31;13:e19145. doi: 10.7717/peerj.19145 (PMC11967419; doi:10.7717/peerj.19145)
Supplement: Supplemental Information 4 — (1) The original image prior to ROI labeling; (2) Images post-ROI labeling; and (3) Label instructions. As the uAI research portal (uRP) is a business intelligence research platform, the computer code is currently unavailable due to commercial interests. We have added a literature to the manuscript (the 23rd article) and attached the full text of the literature, which provides an in-depth introduction to this research platform as well as the data processing procedure. [file peerj-13-19145-s004.zip › Supplemental File/Description of labeling.docx]

Requirements for labeling：

a. The thinnest layer sequence should be prioritized for annotation, and layers with thickness exceeding 5mm are not allowed.

b. When outlining the pneumonia, the lesion should be enlarged to occupy the main field of view size, and then outlined freely.

c. The lesion should be outlined completely on each layer, and there should be no missing or incomplete layers.

d. The contour should fit the edge of the lesion, and there should be no obvious over-labeling, mislabeling, or missing labels. The contour should not extend beyond the lung field (both sides of the rib margin and mediastinal margin), and no labels should be placed on normal lung tissue (large blood vessels, trachea, etc.) except in cases where the boundary cannot be confirmed.

e. Small lesions should not be missed.

f. Pleural effusion, lymph nodes, and calcification in non-pneumonia regions do not need to be labeled.

g. For adjacent lesions that are separated by a clear interval, they should be separately outlined.

h. When different lung lobe lesions are difficult to distinguish, they may be outlined together.

i. When the contour is unclear, it should be outlined to include as much of the lesion area as possible while adhering to the principle of not over-labeling; however, when the contour is unclear due to respiratory motion artifacts, the uncertain area caused by the artifact should be avoided as much as possible.

j. When blood vessels and trachea pass through the lesion, the part of the blood vessels and trachea passing through the lesion may be outlined. However, when the blood vessels and trachea are close to the lesion and can be clearly separated, they should not be outlined.
