# Supplementary material for: Computed tomography-based radiomic features combined with clinical parameters for predicting post-infectious bronchiolitis obliterans in children with adenovirus pneumonia: a retrospective study
Source: PeerJ. 2025 Mar 31;13:e19145. doi: 10.7717/peerj.19145 (PMC11967419; doi:10.7717/peerj.19145)
Supplement: Supplemental Information 4 — (1) The original image prior to ROI labeling; (2) Images post-ROI labeling; and (3) Label instructions. As the uAI research portal (uRP) is a business intelligence research platform, the computer code is currently unavailable due to commercial interests. We have added a literature to the manuscript (the 23rd article) and attached the full text of the literature, which provides an in-depth introduction to this research platform as well as the data processing procedure. [file peerj-13-19145-s004.zip › Supplemental File/uRP: An integrated research platform for one-stop analysis of medical images.pdf]

frontiers | Frontiers in Radiology

TYPE Original Research
PUBLISHED 18 April 2023
DOI 10.3389/fradi.2023.1153784

Check for updates

OPEN ACCESS

EDITED BY
Ajay Mittal,
Panjab University, India

REVIEWED BY
Guotai Wang,
University of Electronic Science and Technology of China, China
Yuan Xue,
Johns Hopkins University, United States

*CORRESPONDENCE
Feng Shi
✉ feng.shi@uii-ai.com
Dinggang Shen
✉ Dinggang.Shen@gmail.com

[†]These authors have contributed equally to this work

SPECIALTY SECTION
This article was submitted to Artificial Intelligence in Radiology, a section of the journal Frontiers in Radiology

RECEIVED 30 January 2023
ACCEPTED 31 March 2023
PUBLISHED 18 April 2023

CITATION
Wu J, Xia Y, Wang X, Wei Y, Liu A, Innanje A, Zheng M, Chen L, Shi J, Wang L, Zhan Y, Zhou XS, Xue Z, Shi F and Shen D (2023) uRP: An integrated research platform for one-stop analysis of medical images.
Front. Radiol. 3:1153784.
doi: 10.3389/fradi.2023.1153784

COPYRIGHT
© 2023 Wu, Xia, Wang, Wei, Liu, Innanje, Zheng, Chen, Shi, Wang, Zhan, Zhou, Xue, Shi and Shen. This is an open-access article distributed under the terms of the Creative Commons Attribution License (CC BY). The use, distribution or reproduction in other forums is permitted, provided the original author(s) and the copyright owner(s) are credited and that the original publication in this journal is cited, in accordance with accepted academic practice. No use, distribution or reproduction is permitted which does not comply with these terms.

uRP: An integrated research platform for one-stop analysis of medical images

Jiaojiao Wu[1†], Yuwei Xia[1†], Xuechun Wang[1†], Ying Wei[1], Aie Liu[1], Arun Innanje[2], Meng Zheng[2], Lei Chen[1], Jing Shi[1], Liye Wang[1], Yiqiang Zhan[1], Xiang Sean Zhou[1], Zhong Xue[1], Feng Shi[1]* and Dinggang Shen[1,3,4]*

[1]Department of Research and Development, Shanghai United Imaging Intelligence Co., Ltd., Shanghai, China, [2]Department of Research and Development, United Imaging Intelligence Co., Ltd., Cambridge, MA, United States, [3]School of Biomedical Engineering, ShanghaiTech University, Shanghai, China, [4]Shanghai Clinical Research and Trial Center, Shanghai, China

Introduction: Medical image analysis is of tremendous importance in serving clinical diagnosis, treatment planning, as well as prognosis assessment. However, the image analysis process usually involves multiple modality-specific software and relies on rigorous manual operations, which is time-consuming and potentially low reproducible.
Methods: We present an integrated platform - uAI Research Portal (uRP), to achieve one-stop analyses of multimodal images such as CT, MRI, and PET for clinical research applications. The proposed uRP adopts a modularized architecture to be multifunctional, extensible, and customizable.
Results and Discussion: The uRP shows 3 advantages, as it 1) spans a wealth of algorithms for image processing including semi-automatic delineation, automatic segmentation, registration, classification, quantitative analysis, and image visualization, to realize a one-stop analytic pipeline, 2) integrates a variety of functional modules, which can be directly applied, combined, or customized for specific application domains, such as brain, pneumonia, and knee joint analyses, 3) enables full-stack analysis of one disease, including diagnosis, treatment planning, and prognosis assessment, as well as full-spectrum coverage for multiple disease applications. With the continuous development and inclusion of advanced algorithms, we expect this platform to largely simplify the clinical scientific research process and promote more and better discoveries.

KEYWORDS
research platform, one-stop, medical image analysis, deep learning, semi-automatic delineation, radiomics

1. Introduction

Medical imaging is widely employed in clinical research to investigate effects on diagnosis, staging, treatment planning, and follow-up evaluations (1–4). Medical imaging contains multiple imaging sequences or modalities, such as magnetic resonance imaging (MRI), computed tomography (CT), and positron emission tomography (PET), providing complementary information (5–8). The processing and quantitative analysis of medical images ensure their clinical utility in a variety of medical applications, from general research to clinical workflows.

Most recently, machine learning- and deep learning-based intelligent imaging analyses have shown enormous advantages in providing consistent and accurate image quantifications in multiple applications, including image segmentation, registration,

classification, etc. (9–12). A series of algorithm architectures and strategies have been developed to meet different requirements. For example, U-Net (13, 14), V-Net (15), and nnU-Net (16) exhibit accurate segmentation performance; affine models (i.e., FLIRT, A-SIFT) (17) and deformable models [i.e., FNIRT, ANTS, VoxelMorph (18), Dual-PRNet (19), LDDMM (20)] assist to image registration; ResNet (21), DenseNet (22) and their variants have attracted much attention in classification tasks. Also, varied attention mechanisms and loss functions have been utilized to optimize the deep learning network and improve its robustness (23–26). The accurate analysis of medical images accelerates the development and upgrading of intelligent algorithms that can be integrated into the software to enable easy-to-use clinical research.

Numerous choices of medical image analysis tools integrating advanced algorithms are available. For example, MATLAB (27), Python (https://www.python.org/), 3D Slicer (28) (https://www.slicer.org/), and Mimics (Materialize, Leuven, Belgium) allow general image processing, while FreeSurfer (https://surfer.nmr.mgh.harvard.edu/), chest imaging platform (CIP, https://chestimagingplatform.org/), and OpenSim (29) are proprietarily applied to the brain, lung, knee joint analyses, respectively. Meanwhile, a number of software is dedicated to a specific modality, such as resting-state fMRI data analysis toolkit (REST) (30) and statistical parametric mapping (SPM, UCL Queen Square Institute of Neurology, London, UK) designed for functional MR images; DtiStudio (31) and medical imaging interaction toolkit (MITK) (32) applied to diffusion images; SenseCare (33) provides a range of artificial intelligence (AI) toolkits for specific clinical scenarios such as lung cancer diagnosis and radiotherapy planning. Overall, the software greatly simplifies image processing and makes it easy for clinicians to understand and use.

However, users still face a series of challenges in using the software to achieve one-stop image analysis. First, complex image analysis requires introduction of multiple software to adapt to the respective modalities and organs, which makes it difficult to integrate information from different modalities organically. Second, different software relies on specific environments (e.g., Windows and Linux) and programming languages (e.g., Python, C++, and R), requiring extensive computer knowledge to be used in practice. Third, the feasibility of integrating the latest AI models into the software to iteratively optimize performance is yet to be assessed. Finally, manual contouring regions of interest (ROIs) (34) is always required in scientific research to serve as the gold standard or to extract quantitative metrics, which is time-consuming and may suffer from low reproducibility and consistency due to intra- and inter-observer variability. Therefore, it is desired to design an integrated platform for one-stop analysis of medical images, which needs to be compatible, advanced, easy to use, extensible, and reproducible. In addition, the ideal platform should offer cloud-based services (public or private) to reduce configuration requirements on the user end and allow multiple clients to work simultaneously. A dedicated data management module is also essential for organizing multiple clinical projects and massive medical data, which allows the integration of large-scale data from multiple centers to develop robust algorithms and facilitate collaborative research.

In line with the trend, we propose a multifunctional platform, called uRP (uAI research portal, https://www.uii-ai.com/en/uai/scientific-research), to perform accurate image processing and analysis on demand. The uRP can satisfy the following requirements: (1) Integrating a variety of algorithms with respect to the image's modality (i.e., MRI, CT, PET), body part (i.e., head, chest, abdomen, pelvis), and processing task (i.e., segmentation, registration, classification), to be suitable for diverse applications; (2) Offering friendly interactive user interface (UI) to make clinicians easy to understand and independently implement complete AI-related research; (3) Possessing the extendable capability to enrich existing modules and ensure reusable and reproducible analysis across clinicians, even hospitals; (4) Achieving automatic or semi-automatic image processing (e.g., delineation) to ensure efficient and accurate analysis; (5) Supporting cloud-based computing services with high concurrency and owing a dedicated data management module.

In the below sections, we present an overview of uRP's architecture and major functional modules, including semi-automatic delineation, deep learning-based image segmentation, registration, and classification, as well as radiomics and statistics. The clinical utility of the uRP is demonstrated by three domain analyses of the brain, pneumonia, and knee joint. Representative use cases are exampled to illustrate some of the outcomes that clinicians have achieved by using the uRP. In addition, the modules of the uRP will continue to be developed and extended, and we prospect future design concepts and directions to achieve a more intelligent platform for scientific research and even clinical uses.

2. Materials and methods

In this article, we propose the uRP to realize the one-stop medical image analysis. The architecture and main modules are shown in the following parts.

2.1. Overview of uRP

From 2018, we began to build the uRP to promote one-stop advanced medical image analysis in the context of integrating AI modules. It is intended to facilitate scientific research for clinicians and is therefore designed as flexible modules that can be used directly, combined, or customized for specific application domains. Here, we start by describing its architecture and key components (Figure 1).

2.1.1. Architecture

The design of uRP architecture takes a modular and layered approach. Internally, the software consists of three layers: (1) The lower level is composed of hardware drivers, such as graphics processing unit (GPU) accelerated using NVIDIA CUDA, and cloud servers, such as Amazon web services (AWS), that efficiently use graphics resources of the host system; (2) At the middle level, there is application programming interface (API), primarily Python and C++, contributing a range of algorithms

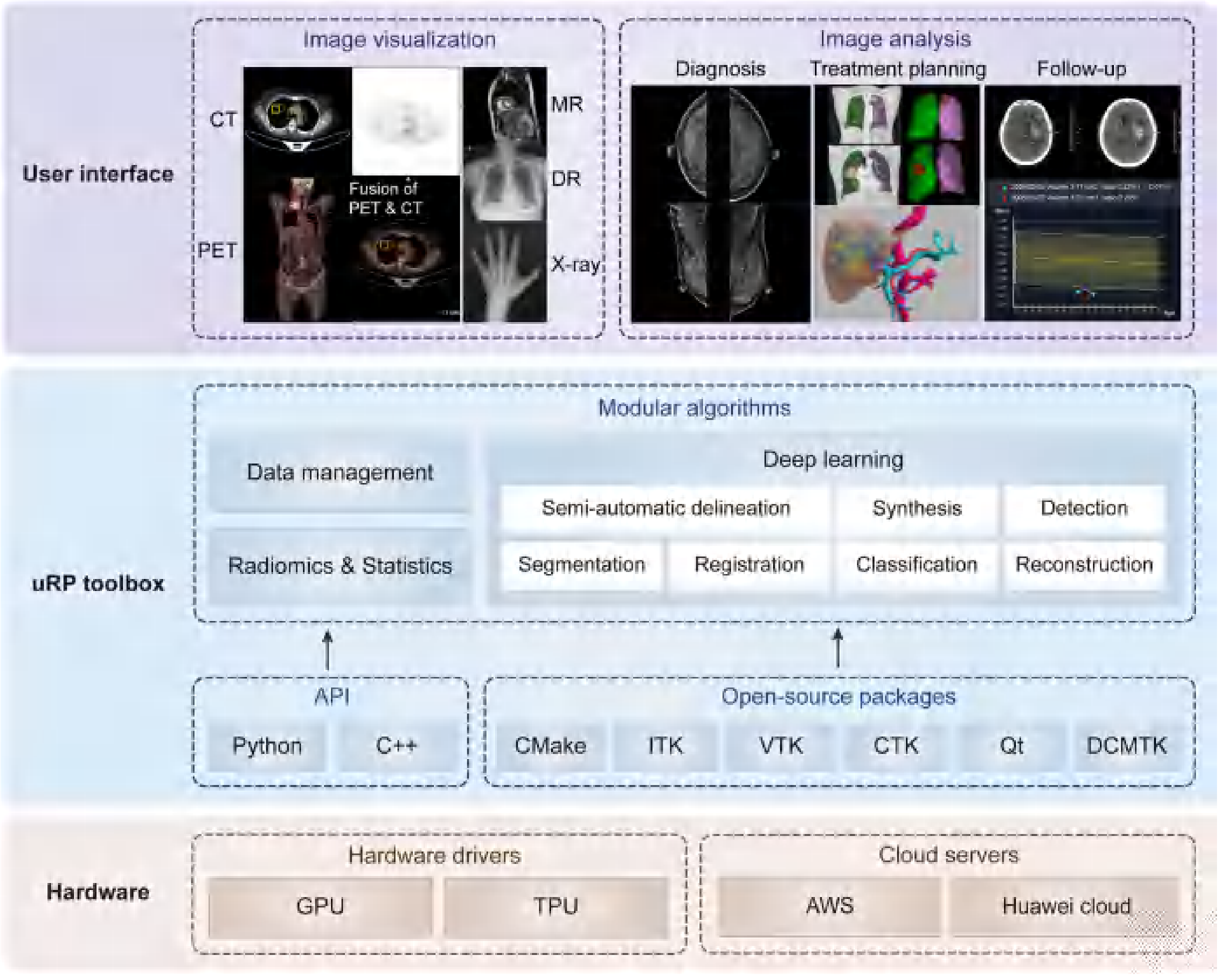

FIGURE 1
Overview of uRP with layered and modular architecture. At the lower level, it involves hardware drivers and cloud servers. At the middle level, it includes the application programming interface (API) and open-source packages to develop algorithms, ensuring versatility for data management, deep learning, as well as radiomics and statistics. At the higher level, it provides a series of user interfaces to perform different application domains covering multiple imaging modalities, full-stack analysis of one disease (i.e., diagnosis, treatment planning, and follow-up), and full-spectrum coverage for multiple diseases.

(e.g., segmentation, registration, classification) and providing higher-level functionality and abstractions. Additionally, a variety of open-source libraries are embedded in the uRP, where the DICOM toolkit (DCMTK) is used to support DICOM format data, and Qt to provide a cross-platform graphical user interface (GUI) framework; (3) The higher level presents UIs of multiple algorithms and builds blocks to the end users for domain-specific analysis (Figure 1). All computational demanding modules can run in parallel on many processors at once, using message passing between processes and/or shared-memory threads.

Since its inception, uRP has been evolving with major architecture, UIs, and functional redesigns. The uRP's version is updated every 2 months, and each release is formally tested on a variety of platform configurations to ensure its stability.

2.1.2. Interactive UI

uRP is a web-based platform supporting GPU cloud computing. It supports plug-ins to deliver task-specific functionality to the user. uRP mainly consists of three functional modules, (1) data management, supporting the upload of imaging data and non-imaging features, extraction of subject information from DICOM tags, and image search based on specific criteria; (2) image processing, including semi-automatic delineation, deep learning-based segmentation, registration, and classification; (3) radiomics and statistics, for classification and regression tasks. Images in DICOM or NIFIT formats from clinical picture archiving and communication system (PACS) or external drives are supported with various imaging modalities, including MR, CT, PET, x-ray, and digital radiography (DR) images. These technical modules can support a variety of applications on demand (Figure 1).

2.2. Semi-automatic delineation

Delineation of ROIs is essential in clinical research as the primary step for quantification and feature extraction. Manual delineation is generally considered as the gold standard, but it is

limited by its tedious, time-consuming, and error-prone characteristics, and thus difficult to achieve high-quality and efficient annotation especially with the massive amount of medical images. In view of this, various automatic delineation algorithms have been developed, including fully automatic and semi-automatic methods. It is worth noting that fully-automatic delineation is convenient but sometimes hard to reach the desired accuracy, while semi-automatic delineation allows human-computer interaction and thus can optimize the results, which is a time-saving alternative to manual delineation. Our proposed uRP platform contains a collection of tools for fully-automatic, semi-automatic, and manual delineation. As shown in Figure 2, a smart annotation tool and a ROI modification tool are integrated into the uRP to assist the delineation process.

2.2.1. Smart annotation tool

The uRP offers several smart annotation tools (SATs) for medical images, including (1) intelligent interactive segmentation and (2) annotation propagation, which enables fast extraction of the target from the complex background and 3D propagation of the annotation.

Intelligent interactive segmentation is a technique that allows the user to adjust the region of interest (ROI) by manipulating seeds. In use, the user firstly draws a rectangle to cover the ROI, where the Canny edge detection algorithm is then performed within the rectangle to generate the target boundary. After that a positive seed is generated at the centroid, and 4 negative seeds are placed at the vertices of the user-defined rectangle, serving as the control points for the boundary (Figure 2A, Supplementary Video 1). We can then update the shape and size of the generated boundary by adjusting the positive / negative seeds. Also, more positive seeds (green) can be added to enlarge the ROI by clicking on the area outside the edge, and negative seeds (red) can be added in the ROI to remove specific regions. Canny's approach is a widely used edge detection method with tweakable parameters and thus suitable to be integrated with a GUI. The algorithm can be divided into the following 5 steps: (1) Use a Gaussian filter to smooth the image and reduce the noise; (2) Calculate the gradient intensity and direction for each pixel point in the image; (3) Edge candidates are identified by applying the non-maximal or critical suppression to the gradient magnitude; (4) Apply double-threshold detection to determine real and potential edges; and (5) Finalize the edge detection by suppressing isolated weak edges (35). The implementation for the Canny edge detection algorithm could be found in the OpenCV library (36).

Another tool is annotation propagation, which applies the current annotation in one slice to its adjacent slices. Specifically, the current annotation serves as the initial mask, and the tool can automatically propagate annotations across entire image frames (Supplementary Video 2). Especially, the user can optionally keep the correct region when the ROI is propagated to other slices and divided into multiple regions. Users can save annotations for following radiomics and deep learning analyses on the uRP, or download them for future review.

2.2.2. ROI modification tool

After labeling the ROI, a variety of specific preprocessing needs to be performed on the mask to meet diverse image analyses. For example, studying tumor microenvironment requires obtaining the peritumor region, whereas studying liver fat requires extracting the ROI by intensity. The uRP offers a morphological modification tool to dilate or erode the selected ROI according to user-defined distances in the x, y, and z directions. For existing ROIs, separation can be performed by user-defined split intervals, connectivity, or minimum size (voxels). It can also merge, intersect, and complement multiple ROIs by performing linear operations on the pixels at each position of the ROI (Figure 2B). When conducting multimodal research, ROI can be duplicated across modalities. The above ROI modification tools can be applied to 2D and 3D ROI, and users can expand research directions with a variety of ROI preprocessing tools available on the platform.

2.3. Deep learning modules

With the advancement of deep learning and its wide application in medical image processing (37, 38), this technology has shown great potential in organ segmentation (39), disease diagnosis (40), etc. Some powerful online trainable deep learning modules are available on the uRP, such as the segmentation module, registration module, and classification module. The network architecture of each module is shown in Figure 3. The segmentation module can segment various organs of the whole body using a cascade coarse-to-fine framework (Figure 3A); the registration module performs unsupervised registration from a moving image to a reference image (Figure 3B), and the classification module can focus on ROI to classify the input image (Figure 3C). These deep learning modules are directly invoked through a simple parameter configuration, which can be flexibly applied to various research scenarios.

2.3.1. Segmentation

The input of the segmentation module can be single- or multimodal 2D or 3D data, where models can be trained to automatically delineate ROIs, such as organs or tumors. The uRP integrates a segmentation toolkit named VB-Net (41, 42). Briefly, we use a V-Net as the backbone for the segmentation task, which consists of a compression path, an expansion path, and skip connections (43). The compression path extracts high-level context information and the expansion path upsamples the signal to recover its original size, where skip connections allow the extracted high-level context information to be fused with fine-grained local information. To reduce model parameters and GPU memory cost, bottleneck layers are added in the down block and up block of the network (Figure 3A). Moreover, a variety of optimization strategies are embedded in the network to improve segmentation performance and extend application scenarios, as described below:

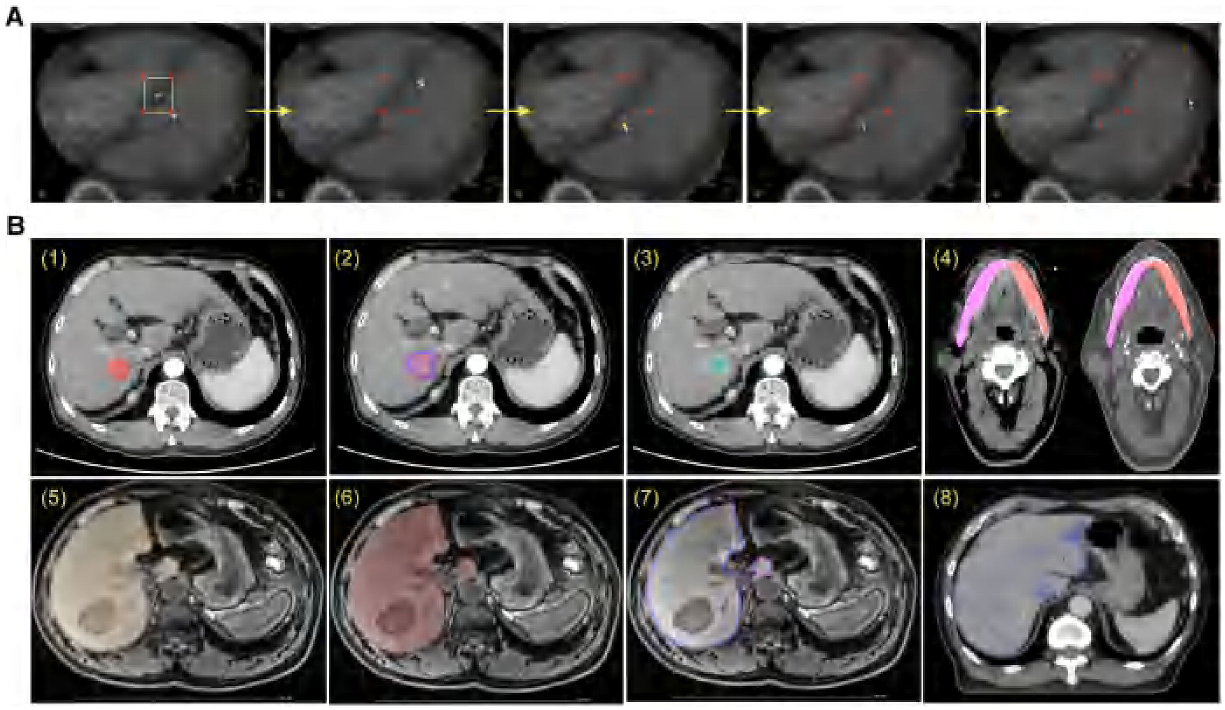

FIGURE 2
Image preprocessing by semi-automatic delineation tool. (A) Smart interactive segmentation. (B) Preprocessing methods of the original ROI (1), including dilation (2), erosion (3), duplication (4), union (5), intersection (6), complement (7), and separation by intensity (8).

(1) Adaptive input module, which adds convolutional layers to large-size images to ensure the network adapts to various input images; (2) A cascade coarse-to-fine strategy, in which the coarse-resolution model aims to localize ROIs in the original image by leveraging the global 3D context, and the fine-resolution model focuses on refining detailed boundaries of ROIs (Figure 3A); (3) Self-attention mechanism, which accelerates network convergence and improves segmentation accuracy; (4) Various loss functions, such as Dice loss, focal loss, and boundary loss, which can effectively constrain the segmented targets in different tasks (41).

Scientific research users can invoke the deep learning segmentation module directly through the configuration file, requiring no coding skills in the process (Supplementary Video 3). The configuration file provides the following functions: (1) setting paths of training data, model storage, and output results; (2) hyperparameters during the training process (e.g., GPU selection, batch size, training epochs); (3) data augmentation (e.g., flipping, rotating, scaling), (4) data preprocessing (e.g., sampling method, cropped size, padding type, normalization); (5) configurations of the loss function, optimizer, and learning rate scheduler; (6) segmentation evaluation metrics (i.e., Dice, average symmetrical surface distance, Hausdorff distance); (7) configurations of networks and strategies, e.g., cascading of different networks and selection of attention mechanisms. The configuration file should be configured in a standard format (a template is provided on the uRP) and called in the training process.

Moreover, the segmentation module integrates more than 100 high-precision organ segmentation models throughout the body. Segmentation of organs at risk using the uRP's segmentation module has shown great advantages in terms of speed (0.7 s vs. 20 s per organ), accuracy (average Dice score 96.6% vs. 84.3%), and robustness (successful rate 98.6% vs. 83.3%) compared to conventional methods (44).

2.3.2. Registration

Image registration is to align a moving image to a reference image, which is a critical procedure in the analyses of multimodal images and longitudinal data (45). The uRP provides both traditional image registration (i.e., rigid, affine transformation) and deep learning-based nonlinear registration. Briefly, the nonlinear registration model consists of a registration network, spatial transform block, and hybrid loss calculation module. A hybrid loss is calculated to strengthen the alignment constraints of different structures, which combines image dissimilarity, deformation regularization, and segmentation dissimilarity with different weights (Figure 3B). The available image dissimilarity metrics include mean square difference (MSD), normalized correlation (NC), mutual information (MI), etc. Image registration can be performed between the images of the same modality or of different modalities; for example, MI is generally selected as the image dissimilarity for cross-modal registration.

Notably, the segmentation results obtained automatically from the segmentation module could also be used in the registration process. In previous studies, organ segmentations served as soft constraint in the loss function to provide auxiliary information in the training of the registration model (46, 47). Compared to

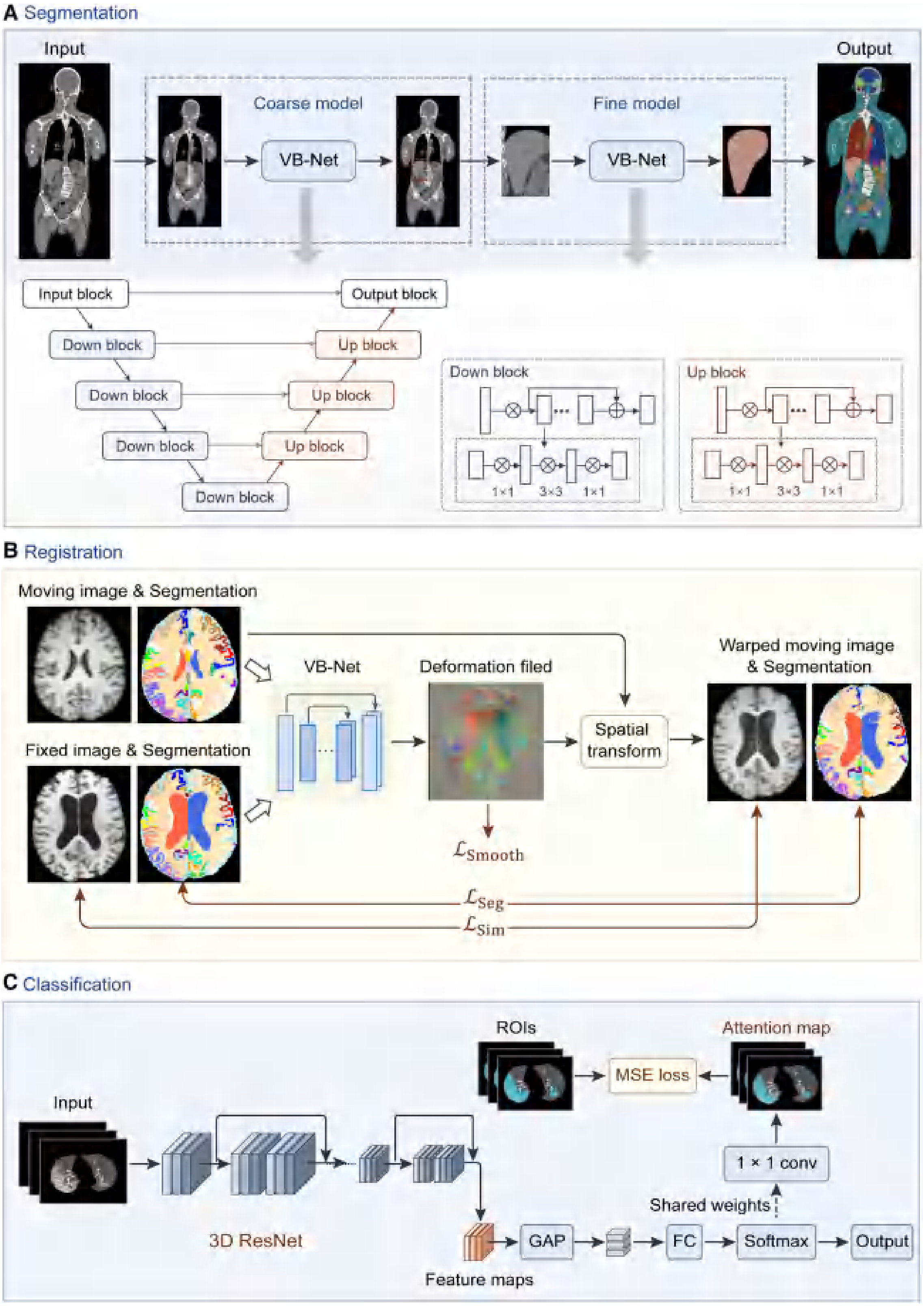

FIGURE 3
Network architectures of deep learning modules in the uRP. (A) Workflow of segmentation network (VB-Net) with a coarse-to-fine strategy, which first roughly locates the target area and then segments the fine boundary of the ROI. (B) Image registration framework with introduced region segmentations as constraints. (C) Classification network from images to features, with ROI attention strategy. GAP, global average pooling; FC, fully connected layer.

using only the intensity image for registration, these studies found that the large deformation can be more readily estimated with the help of the segmentation result.

2.3.3. Classification

The uRP integrates a classification module and can be used for two-class and multi-class tasks. ResNet (48) is used as the classification backbone and optimized by several strategies: (1) an online attention module as CAM (49) and Grad-CAM (50), that ensures the network to focus on ROIs and increases the model interpretability (Figure 3C); (2) a balanced sampling mechanism, which can alleviate the imbalanced distribution of the input data; (3) different sampling methods for inputs; (4) various loss functions, e.g., focal loss (51), Ap loss (52), and CAM loss. A lot of classic and mature classification networks such as DenseNet (53) and EfficientNet (54) have been embedded in the uRP. Moreover, the classification module can be flexibly invoked via a configuration file, similar to the segmentation module.

2.4. Radiomics and statistics

Radiomics is a quantitative image analysis technique through extracting quantitative features from medical images, that aims to

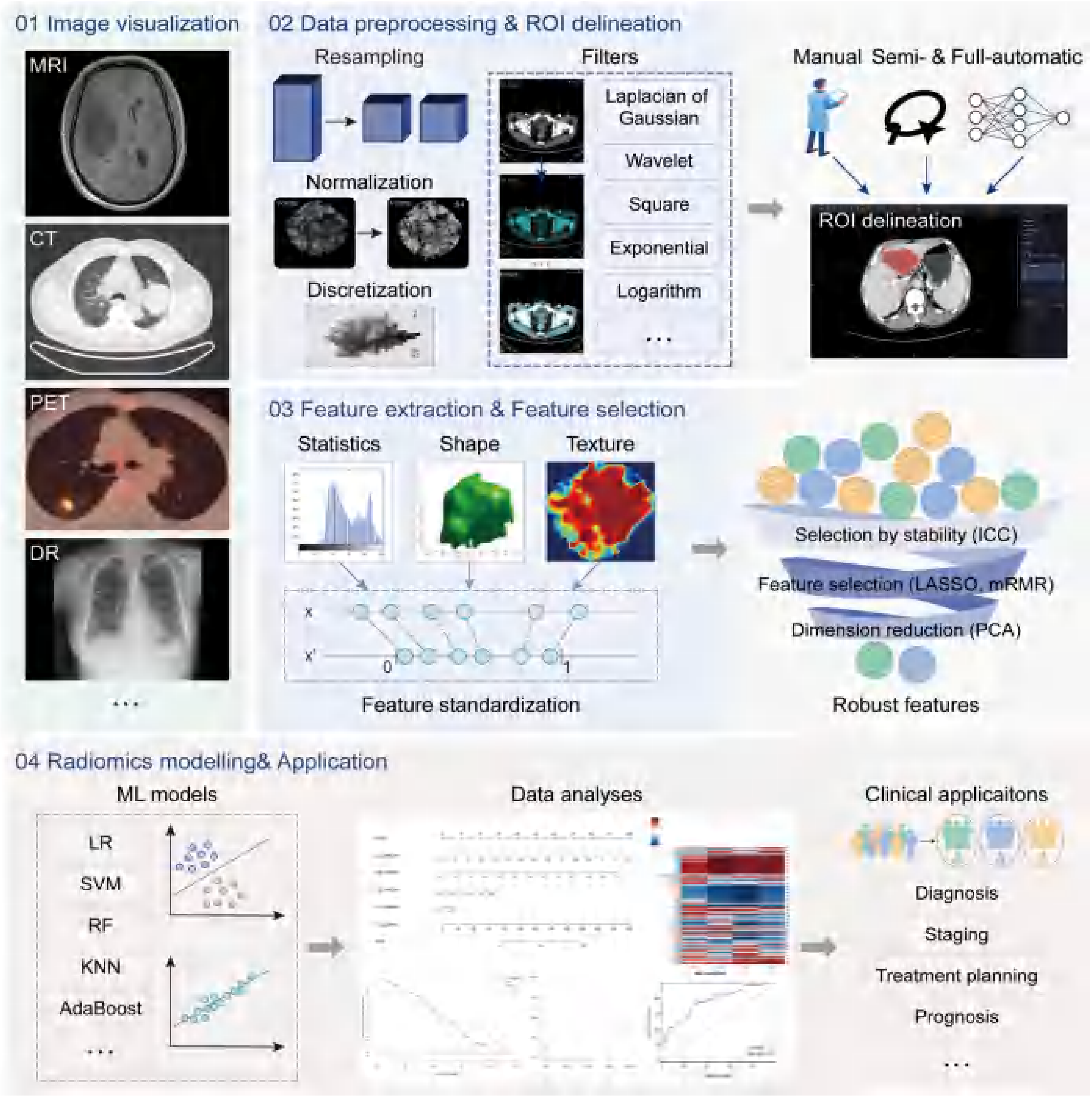

FIGURE 4
The radiomics analysis workflow. The radiomics analysis module supports four main functions, including (1) image visualization for multi-modal images, (2) data processing and region of interest (ROI) delineation, (3) feature extraction and selection, and (4) model construction and evaluation. The above analysis workflow can be used in a variety of clinical applications.

link large-scale data mining of images with clinical and biological endpoints (55). The uRP implements a one-stop analytic pipeline of radiomics, providing clinical researchers with a simple UI for image visualization, image processing, feature analysis, model construction and evaluation, and statistical analysis (Figure 4).

2.4.1. Data preprocessing and grouping

Previous studies have shown that radiomics features are sensitive to variations in gray level, pixel size, and slice thickness of images (56–59). However, it is difficult to standardize parameters during image acquisition for all patients in a clinical setting. The platform supports a variety of normalization algorithms to normalize image signal intensity, such as mean_std, max_min, and center_width. And it resamples the image and ROI mask to specified pixel spacing to achieve a standardized variable pixel size and slice thickness by resampling algorithms, such as the nearest neighbor, linear interpolation, and B-spline interpolation. A pre-defined bin number of 64 is used for all analyses. In image processing and feature calculation, we follow the guidelines of the imaging biomarker standardization initiative (IBSI) (60).

The uRP supports three data grouping methods, namely customized grouping, random grouping according to proportion, and cross-validation grouping. The training set is used for feature selection and model construction, and the model performance is evaluated on the testing set.

2.4.2. Feature extraction and standardization

After preprocessing the images, a total of 2,264 radiomics features can be automatically extracted from each ROI (Supplementary Table S1). The first-order statistics include 18 features that reflect the quantitative depiction of the distribution of voxel intensity in medical images. The shape-based features include 14 features that reflect the shape and size of a region. The textural features include 21 gray level co-occurrence matrix (GLCM) features, 16 gray level run length matrix (GLRLM) features, 16 gray level size zone matrix (GLSZM) features, 5 neighboring gray-tone difference matrix (NGTDM) features, and 14 gray levels dependent matrix (GLDM) features, which quantify regional heterogeneity differences. Additionally, the derived images are obtained by applying 24 filters (box mean, additive Gaussian noise, binomial blur, curvature flow, box-sigma, normalize, Laplacian sharpening, discrete Gaussian, mean, speckle noise, recursive Gaussian, shot noise, LoG (sigma: 0.5, 1, 1.5, 2), and wavelets (LLL, LLH, LHL, LHH, HLL, HLH, HHL, HHH)), and are used to extract first-order statistics and textural features (2,160 derived features). Most features defined in the uRP conform to feature definitions described in the IBSI (60).

To ensure the clinical utility of the model, features beyond radiomics should also be considered to improve the model's generalizability, such as demographic information and biological data. Considering that radiomics features and clinical features have different ranges, feature standardization algorithms are also provided in the uRP, such as z-score_scaler, min_max_scaler, quantitle_transformer, yeojohnson_transformer, boxcox_transformer, L1_normalization, L2_normalization, and max_abs_scaler (61).

2.4.3. Feature selection and model construction

The feature selection module is used for feature selection or dimension reduction to improve model performance. It includes variance thresholding for removing low variance features, SelectKBest for removing high p-value features, as well as least absolute shrinkage and selection operator (LASSO) for sparse feature selection. It also includes factor analysis, independent component analysis (ICA), linear discriminant analysis (LDA), principal component analysis (PCA), and more than 10 algorithms for dimension reduction to get fewer new features formed by original features.

Based on the selected features, various machine learning-based models can be constructed for classification or regression tasks. Our proposed uRP integrates 13 machine learning algorithms, including adaptive boosting (AdaBoost), bagging decision tree, decision tree, Gaussian process, gradient boosting decision tree (GBDT), K-nearest neighbors (KNN), random forest (RF), logistic regression (LR), extreme gradient boosting (XGBoost), stochastic gradient descent (SGD), support vector machine (SVM), quadratic discriminant analysis (QDA), partial least squares-discriminant analysis (PLS-DA), and allows for hyperparameters adjustment. The nomogram model is also included in this module, which combines radiomics and clinical factors to facilitate the clinical utility of predictive models. It is necessary to first calculate the score of each predictive variable, obtain the total point, and then find the probability of the disease outcome corresponding to the total score.

2.4.4. Evaluation metrics

To evaluate model performance, the uRP provides two sets of quantitative metrics for classification and regression models.

(1) Classification model: The platform can automatically generate receiver operating characteristic (ROC) curves and calibration curves in the training and validation cohorts, and calculate multiple metrics, such as the area under the ROC curve (AUC, with 95% confidence interval), F1 score, precision, sensitivity, specificity, and accuracy. It also provides a visual representation of the confusion matrix and supports the comparison of multiple models. In addition, the histogram, box chart, violin chart, correlation analysis heatmap, and clustering analysis heatmap are optionally plotted to establish the relationship between features. For clinical applications, the decision curve and clinical impact curve can be plotted to assess the clinical usefulness of models by quantifying net benefits at different risk thresholds.

(2) Regression model: The platform automatically generates prediction curves and scatter plots to visualize the results, and calculates mean absolute error (MAE), mean absolute error (MSE), R-squared, and Pearson correlation coefficient for regression model evaluation.

3. Results

To illustrate the clinical utility of the uRP, we list three domain analyses (i.e., brain, pneumonia, and knee joint) and representative use cases.

3.1. Specific domain analysis

It is worth noting that uRP can be extended and applied to a variety of designated applications (Figure 1), e.g., brain structural analysis, pneumonia analysis, and knee joint analysis. In this section, we focus on these three specific scenarios to describe the versatility and scalability of uRP, covering segmentation, quantitative analysis of ROI, classification for disease prediction, and prognosis, where image analysis modules are integrated into sequential to form an automatic analysis pipeline.

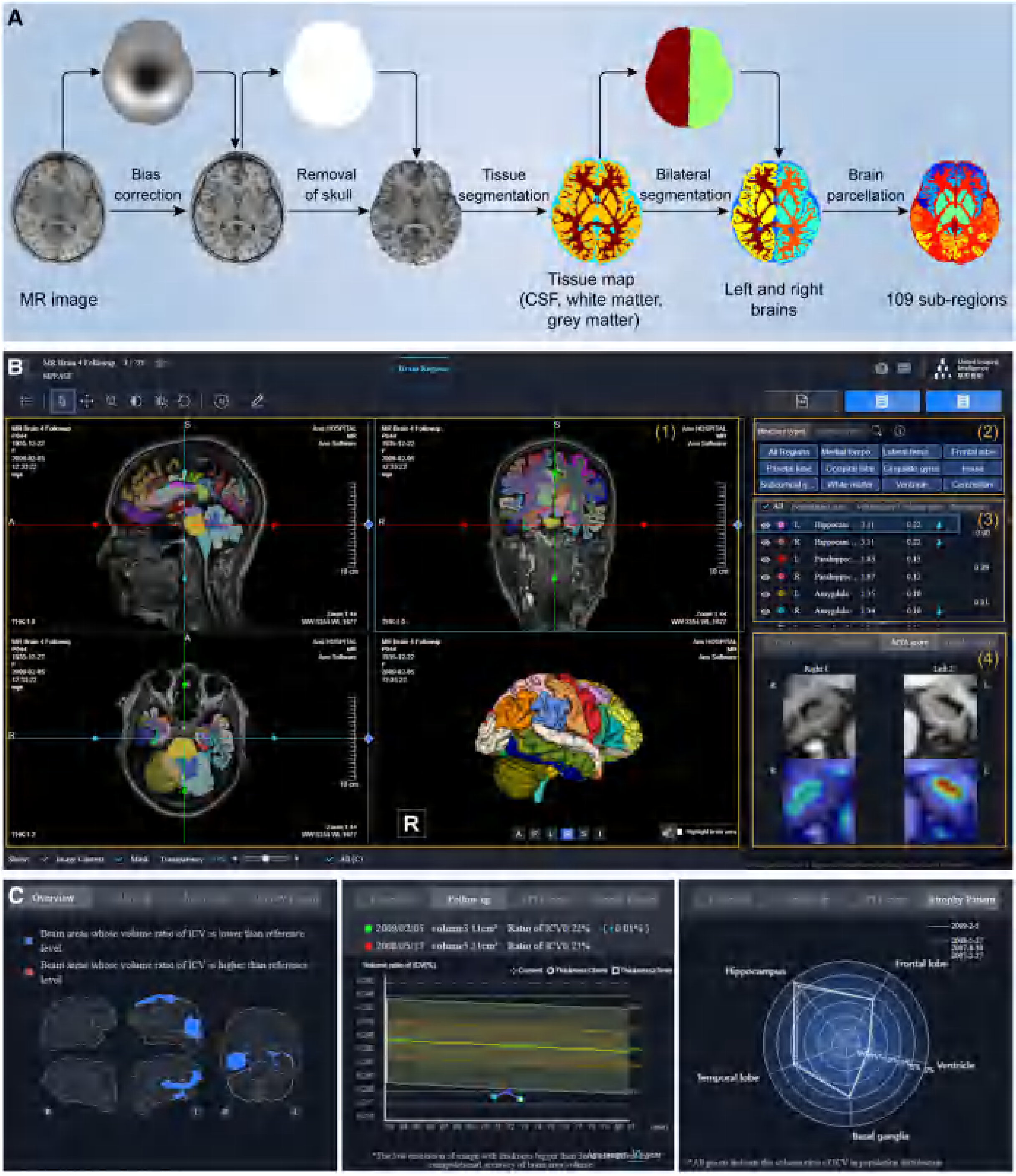

FIGURE 5
One-stop brain analysis module integrated into the uRP. (A) Segmentation pipeline of brain structure, including the bias field correction, removal of the skull, tissue segmentation of white matter, gray matter, and cerebrospinal fluid (CSF), bilateral segmentation, and parcellation of 109 sub-regions. (B) The user interface (UI) of brain analysis, including (1)–(2) image visualization of the specific region or disease, (3) quantitative volume analysis, and (4) MTA-scale score. (C) UI showing abnormal regions and follow-up analysis.

3.1.1. Brain analysis

Neuroimaging shows tremendous potential in the early diagnosis of neurodegenerative diseases (62, 63), in which structural imaging (MRI) serves as a foundation to provide brain tissue and parcellation information (Figure 5).

First, uRP can handle high-resolution MR images and hierarchically segment brain structures (Figure 5A). The workflow mainly involves: (1) the bias field correction, (2) removal of the skull, (3) tissue segmentation of white matter, gray matter, and cerebrospinal fluid (CSF), (4) bilateral segmentation, and (5) parcellation of 109 sub-regions. A total of 109 sub-regions includes 22 temporal lobe structures, 20 frontal lobe structures, 12 parietal lobe structures, 8 occipital lobe structures, 8 cingulate gyrus structures, 2 insular structures, 12 subcortical gray matter structures, cerebral white matter structures, ventricles, cerebellum, and other structures (64). To emphasize, this segmentation process depends on VB-Net, achieving efficient, precise, and end-to-end segmentation of multiple sub-regions. The model was trained on T1 images of 1,800 subjects and tested on 295 subjects with an average Dice of 0.92, where the images were acquired from the Consortium for Reliability and Reproducibility (CoRR) dataset (65) and Chinese brain molecular and functional mapping (CBMFM) project (66). Based on the segmentation results, the volume, volume ratio of each sub-region, and the asymmetry index of paired sub-region are calculated quantitatively and compared to the relevant parameters from the gender- and age-matched normal dataset (Figure 5B). Abnormal brain sub-regions are identified, where those below the 5th percentile of the normal range are considered likely to be abnormally atrophic, and those above the 95th percentile are considered abnormally enlarged (Figure 5C). On the other hand, the medial temporal lobe atrophy (MTA) score, also known as Schelten's scale, is developed with an AI model for automated assessment of the hippocampus atrophy status (67). The score ranges from 0 to 4, in which the higher the score, the more severe the hippocampal atrophy (Figure 5B). It should be noted that the segmentation and computation of the 109 sub-regions take less than 1 min.

Based on this, uRP holds enormous ability in the follow-up data analysis, i.e., (1) comparing volume changes of ROIs over time to explore pathological progression of neurodegenerative diseases; (2) constructing AI models to predict the odds of other diseases such as Parkinson's disease (PD) or mental diseases for early diagnosis and early intervention; (3) promoting the brain functional analysis with established structural ROIs, as well as fiber connectivity analysis in diffusion tensor imaging (DTI) (Figure 5C).

3.1.2. Pneumonia analysis

With the worldwide spread of coronavirus disease (COVID-19), the early diagnosis and prognostic analysis of pneumonia have become an urgent need, which inspires a large number of related researches to serve the clinic (68–71). CT has been popularly used to monitor pneumonia's progression and measure the disease severity (72–74). Based on chest CT scans, three important issues need to be explored: (1) the location of the pneumonia infection, (2) the severity of the infection, and (3) the etiology of the disease. uRP meets these requirements by integrating segmentation, classification, and registration algorithms (Figure 6A).

The first step is to locate the infected lesions. First, the whole lung is obtained by embedded VB-Net, followed by bilateral segmentation (75). Then, the left lung is segmented into 2 lung lobes (superior and inferior lobes), while the right lung is segmented into 3 lung lobes (superior, middle, and inferior lobes). Afterward, 5 lung lobes are then finely segmented into 18 bronchopulmonary segments (Figure 6B). Infected lesions are also auto-contoured in this process, and can be visualized from the UI. Noted that the human-in-the-loop strategy is designed to iteratively update VB-Net to address the problem of limited annotated data (73). To be specific, an initial segmentation model based on a small amount of delineated data is applied to the new data, and segmentation results are manually corrected and then fed into the model, so that a more robust model will be trained through 3~4 iterations, greatly improving the efficiency of delineation.

Following the segmentation, a diverse set of handcrafted features are calculated to quantitatively assess the severity of the pneumonia infection (75), including (1) 26 volumetric features—the volume and percentage of infections in each lobe and pulmonary segment, (2) 31 numeric features—the number of infected lobes and pulmonary segments, (3) 32 histogram features—the histogram distribution of CT intensity, (4) 7 surface features—the surface area of infections and lung boundary. A total of 96 location-specific features are displayed in the UI to reflect the severity of pneumonia infection. In addition, follow-up data can be registered with previously acquired images to extract changes in infection-specific features to monitor the progression of pneumonia and to accurately determine the severity (Figure 6C).

Importantly, uRP can also distinguish different types of pneumonia and predict possible pneumonia causes *via* uRP's built-in classification algorithms. Based on the segmented masks and extracted features, dual-sampling attention 3D ResNet is used to diagnose COVID-19 from community acquired pneumonia (CAP) (76). Moreover, masks, handcrafted features, as well as radiomics features can be used to classify the cause of pneumonia (e.g., viruses, fungi, and bacteria), and to report the corresponding probabilities.

Therefore, uRP-based pneumonia analyses involve automatic segmentation of infected lesions, extraction and visualization of quantitative metrics, and classification of different types of pneumonia, which largely accelerates scientific research on pneumonia.

3.1.3. Knee joint analysis

Knee osteoarthritis (OA), known as a degenerative joint disease, results from the wear, tear, and progressive loss of articular cartilage, which may eventually lead to disability (77). The severity staging of knee OA should be carefully taken into consideration for the treatment, which relies on a range of morphological parameters, including the volume and thickness of

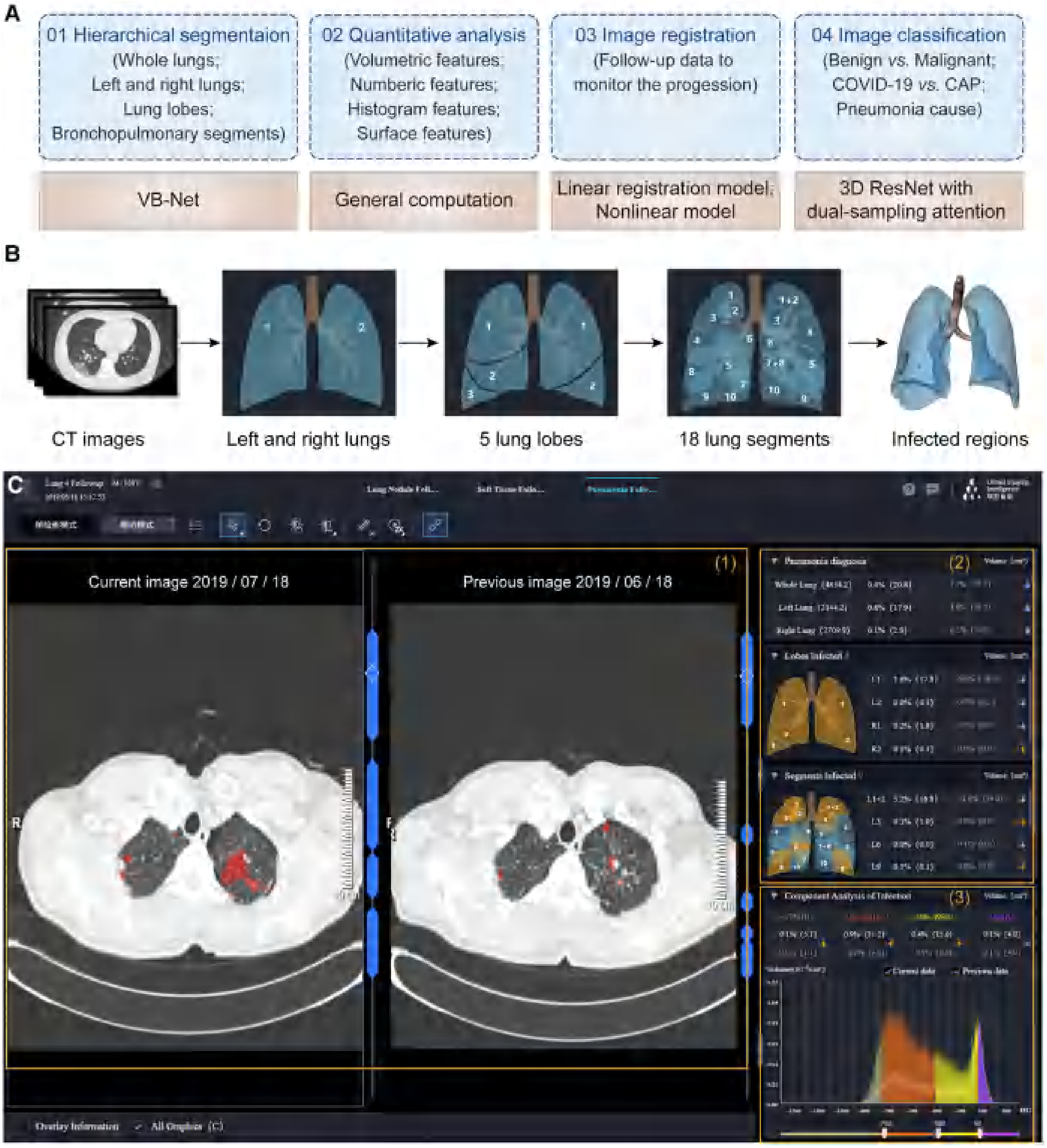

FIGURE 6
One-stop pneumonia analysis module integrated into the uRP. (A) Schematics of pneumonia analysis, including segmentation, computation, registration, and classification. (B) Hierarchical segmentation of lungs and infected lesions. (C) The user interface of pneumonia analysis on the uRP, including (1) image visualization to compare images from two-time points, (2) quantitative analysis for pneumonia diagnosis and severity assessment, and (3) histogram analysis of CT intensity distribution of images for comparison.

articular cartilage, and minimal joint space width (mJSW) (78). To benefit clinical practice, uRP implements a complete analysis pipeline that automatically segments knee tissues and calculates morphological metrics.

At first, a cascade coarse-to-fine strategy is applied to obtain fast and accurate segmentation results (Figure 3A). Through two 3D-VNet segmentation models, multiple knee joint tissues are accurately segmented, including bones (i.e., femoral bone, tibial bone, patella bone), cartilages (i.e., femoral cartilage, tibial cartilage, patella cartilage), as well as menisci, and visualized in the UI of uRP (Figure 7A).

At the same time, a series of morphological parameters are automatically calculated from corresponding segmented masks (79), including (1) volumes of cartilages, menisci, and cartilage damage, (2) the mean thicknesses of cartilages, (3) the medial and lateral mJSWs, calculated with the minimal Euclidean distance

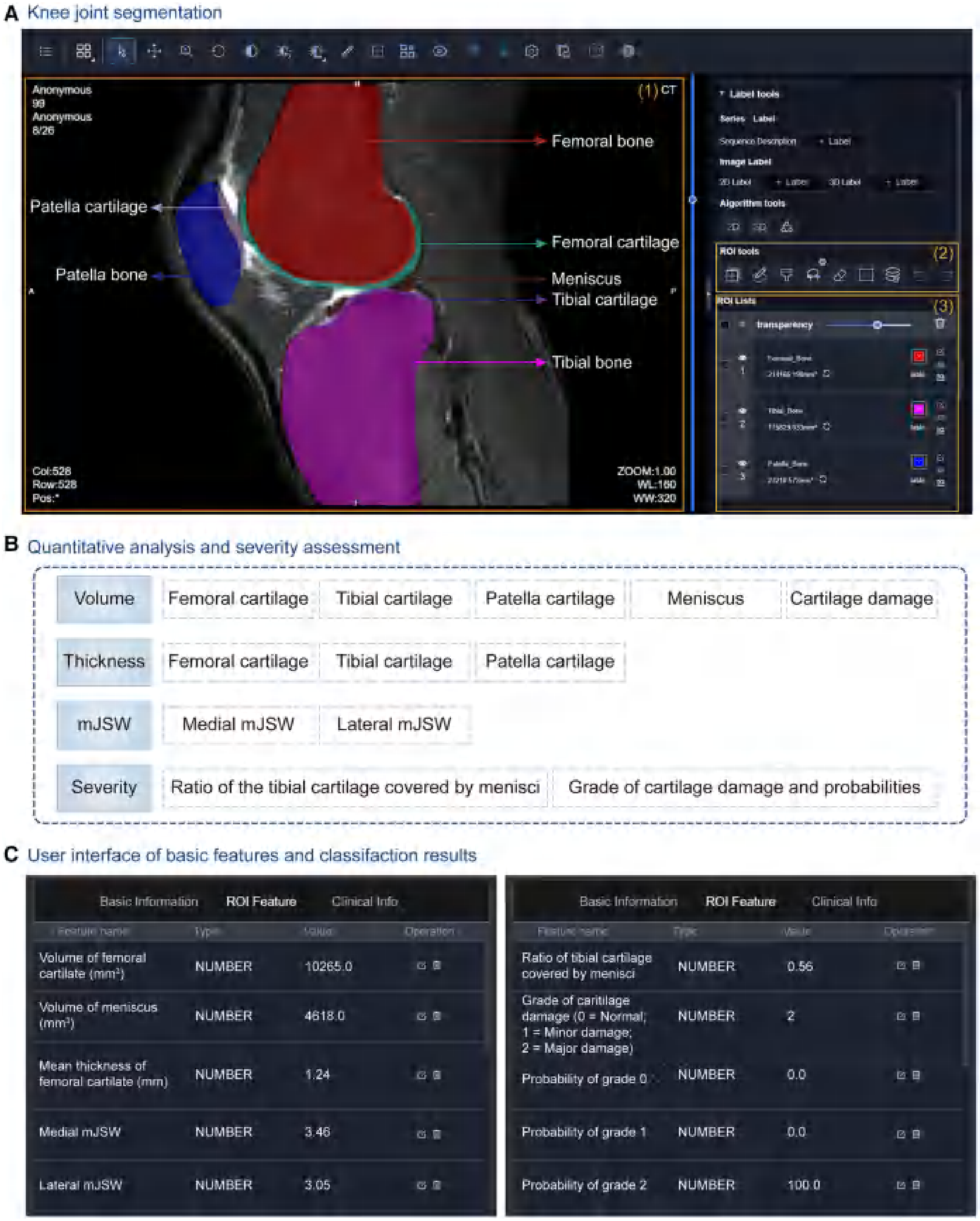

FIGURE 7
Knee joint analysis module integrated into the uRP. (A) The user interface (UI) of segmentation results of the knee joint, in which multiple knee joint tissues are segmented including femoral bone and cartilage, tibial bone and cartilage, patella bone and cartilage, as well as menisci. (B) Quantitative metrics calculated for knee osteoarthritis (OA) diagnosis, i.e., volume, thicknesses, minimal joint space width (mJSW), and severity assessment. (C) UI showing quantified features and classification results for OA severity grading.

between the femoral and tibial surfaces, (4) the severity assessment—the ratio of the tibial cartilage covered by menisci, the grade of cartilage damage and corresponding probability (Figure 7B). Severity grading is also performed by a classification algorithm (Figure 7C). All the above results can be viewed from the uRP, which is helpful for clinicians to make a quick diagnosis of knee OA.

Overall, the knee joint analysis module on uRP can handle MRI images in a fully automatic manner, in which knee joint tissues are segmented and key features (i.e., volume, thickness, mJSW) are calculated to identify the severity of knee OA, thus guiding the optimal treatment.

3.2. Use cases

To illustrate the utility of the uRP, we example several use cases and organize them into the following four parts:

3.2.1. Segmentation use

The automatic segmentation module on the uRP has been used in many medical scenarios, for example, to diagnose COVID-19 infections. Chest CT scans of 549 patients were collected from Shanghai Public Health Clinical Center and several other hospitals, and were automatically segmented and quantified the infected regions throughout the lungs (80). Researchers used the pneumonia analysis module of the uRP, yielding a Dice similarity coefficient of 91.6% ± 10.0% for the segmentation of infected areas, and a mean estimation error of the infected percentage of 0.3% for the whole lung on the validation dataset (300 patients). Besides, to predict the severity of COVID-19 patients, quantitative features of 5 lung lobes and 18 bronchopulmonary segments were calculated and used to construct a classification model based on the SVM algorithm. The best accuracy of severity prediction was 73.4% ± 1.3%, which demonstrated that uRP's pneumonia analysis module owned good performance on patient severity prediction.

3.2.2. Classification use

The image classification module built into the uRP has been experimentally explored in various applications such as disease diagnosis, risk classification, and treatment selection. Gastrointestinal stromal tumors (GISTs) are mesenchymal neoplasms with variable malignant potentials (81). In clinics, accurate preoperative risk classification is important for surgical resection and adjuvant treatment (82). Researchers from Shandong Provincial Hospital and the Affiliated Hospital of Qingdao University collected contrast-enhanced CT images and clinicopathological characteristics from 733 patients and the goal was to develop a model for predicting the GISTs risk stratification (83). A deep learning model with an attention mechanism was constructed on the uRP's classification module to divide patients into three categories (i.e., low-malignant, intermediate-malignant, and high-malignant). The obtained AUCs were 0.90, 0.80, and 0.89 on the testing sets for low-malignant, intermediate-malignant, and high-malignant GISTs, respectively. Therefore, this multi-center study demonstrated that the quantitative CT and deep learning-based approach can be an objective means of predicting the risk stratification of GISTs.

3.2.3. Registration use

Image registration is widely needed for the analyses of multimodal images and longitudinal data. For example, precise registration of dynamic contrast enhanced MR images (DCE-MEIs) with pre-contrast images can be used to obtain accurate subtraction images, which helps to better differentiate the true enhancement of residual viable tumors from coagulative necrosis. In our recent study, Qian et al. collected 3D liver DCE-MRI series from 97 patients, with each series including pre-contrast T1-weighted, post-contrast T1-weighted scan at the arterial phase, and post-contrast T1-weighted scan at the portal venous phase (84). To overcome the intensity enhancement (due to the contrast agent) and spatial distortions of the liver, the cascade registration framework integrated into uRP was used to register the post-contrast images to the pre-contrast images. The registration performance of the proposed method was compared with the traditional registration method SyN in the ANTs toolkit, and the results demonstrated that the proposed framework embedded in the registration module of uRP owned a comparable performance and significantly improved efficiency.

3.2.4. Radiomics use

The radiomics module is particularly suitable for image-based classification and regression tasks, deployed and used well in multiple centers. Radiomics analysis was conducted in the Fourth Affiliated Hospital of Harbin Medical University to discriminate acute myocardial infarction from unstable angina (85). A total of 210 patients with coronary computed tomography angiography (CCTA) images were retrospectively collected and randomly divided into the training and validation cohorts. Following the workflow of radiomics analysis in uRP, three vessel-based pericoronary adipose tissue (PCAT) radiomics features and fat attenuation index (FAI) were extracted from CCTA images, and then selected features were used to construct the classification model. Results demonstrated that the combined model achieved superior performance with AUC values of 0.97 and 0.95 for training and validation cohorts, respectively. The Affiliated Hospital of Southwest Medical University applied a radiomics model to predict the T stage, perineural invasion, and microvascular invasion of extrahepatic cholangiocarcinoma (CCA). This retrospective trial included 101 CCA patients scanned with four MR images, including T1-weighted imaging (T1WI), T2-weighted imaging (T2WI), diffusion-weighted imaging (DWI), and apparent diffusion coefficient (ADC) map. Radiomics features were extracted from four MR images, followed by dimension reduction, and selected features were used to construct three classification models corresponding to the three tasks. The AUC values of models in the testing cohort for predicting T stage, perineural invasion, and microvascular invasion were 0.962, 1.000, and 1.000, respectively (86). Similarly, researchers from Zhongshan Hospital constructed a multi-parametric radiomics nomogram for predicting the microvascular invasion (MVI) based on multiple MR sequences from 130 patients pathologically confirmed with intrahepatic CCA. The nomogram incorporating tumor size, intrahepatic duct dilatation, and the radiomics model, achieved good prediction performance with AUC values of 0.953, 0.861, and 0.819 in the training, validation ($n = 33$), and time-independent testing cohorts ($n = 24$), respectively (87).

4. Discussion

Over the years, uRP has gained broad acceptance within the medical image analysis community, which can be attributed to its breadth of functionality, extensibility, and cross-platform portability.

4.1. Extendable modules

One key strength of uRP is the modularization for customized extensibility, where plug-ins can be designed for specific purposes, and can be freely combined to accomplish complex analyses, suitable for a variety of scenarios. uRP's extensible structure means that new functionality can be integrated on top of the existing platform, rather than being created from scratch, showing significant advantages over monolithic software. The modular design benefits users in several ways:

(1) Reusability: uRP has integrated multiple algorithms that are embedded into axiomatic building blocks and are invoked for specific analysis workflows. An algorithm may participate in many tasks and perform a similar function, meaning that advanced techniques can be reused in new research areas. In addition to existing algorithms, new algorithms can also be developed and integrated into the uRP, for example, transfer learning, transformer networks, etc., to meet clinical needs. Besides, uRP can be extended with third-party software (i.e., 3D Slicer) to import historical annotation data or clinical information.

(2) Reproducibility: The design of plug-ins of uRP follows the criteria of standardization and interoperability, which can be easily shared among research groups, thus minimizing the need for duplication and facilitating reproducibility and consensus building. uRP collects detailed parameters set by the user and generates a report summarizing quantitative metrics and results so that a study can be replicated by different researchers or even different institutions. Equally important, uRP can be used for data management and fair data repositories are essential for reproducible research. Meanwhile, uRP owns reproducibility at scale, e.g., producing high-dimensional radiomics features for ROI in each image or applying the same quantitative analysis in high-throughput images.

(3) Community: uRP has an active community of more than 50 hospitals in China. The feedbacks provided by users fuel improvements of uRP, especially the development of innovative algorithms for clinical needs.

4.2. Advantages

The uRP platform has shown great potential for one-stop image analyses in multiple scientific researches. There are several other platforms for image analysis, such as 3D Slicer (28), SenseCare (33), MITK (32). The proposed uRP has several advantages. (1) uRP can provide cloud-based services allowing for high user concurrency, a data management module to facilitate collaborations, and batch processing capability for efficient analysis. (2) Some of these platforms are for clinical diagnostic and treatment planning usages, with modules such as lung cancer diagnosis, radiotherapy planning for head and neck cancers. uRP is designed for clinical scientific research, supporting image analysis and research idea validation. (3) The uRP integrates machine learning algorithms and statistical analysis methods to provide a more powerful analytical tool for clinical research. (4) The uRP also offers smart annotation tools for medical images, to remedy the time-consuming manual annotation process that serves as a prerequisite for ROI based analysis.

4.3. Outlook

Although uRP already has many applications as a one-stop medical image analysis platform, some issues are still to be addressed. Firstly, uRP currently focuses on the analysis of radiological images, while other types of images could be future supported, such as those from pathology. Secondly, most existing applications are oriented towards the adult population, while applications specific for the fetal, infant, and children are needed. We have only two applications for now, i.e., infant brain segmentation, skeletal age prediction, and would be further increased in the future. While uRP cannot meet all the scientific needs, it is fortunately a dynamic software that evolves together with the new scientific research derived from clinical problems. In the future, we will continue to develop more tools and domain-specific methods, including algorithms, statistics, as well as radiomics, to improve the efficiency, accuracy, robustness, and generalization of one-stop analysis. Represented by ChatGPT, generative AI is a hot research topic for now. It would bring many improvements to the current scientific research, such as better integrating multi-omics data to assist clinical diagnosis and prognosis assessment. We are also exploring the possibility of integrating generative AI in the platform at suitable scenarios (88). We anticipate that the uRP can be applied to diverse domains covering an increasing number of analytic pipelines for diverse pathological diseases.

5. Conclusion

In summary, uRP is a one-stop medical image analysis software for scientific research, and it not only supports versatile visualizations, but also provides advanced functionality such as automatic segmentation, registration, and classification for a variety of application domains. More specifically, it has three major merits, (1) advanced built-in algorithms (>100) applicable to multiple imaging modalities (i.e., CT, MR, PET, DR), diseases (i.e., tumor, neurodegenerative disease, pneumonia), and applications (i.e., diagnosis, treatment planning, follow-up); (2) an iterative deep learning-based training strategy for fast delineation of ROIs of large-scale datasets, thereby greatly saving

clinicians' time and obtaining novel and more robust models; (3) a modular architecture with customization and extensibility, where plugins can be designed for specific purposes. As a result, it will be necessary to investigate and develop new algorithms and strategies to expand application domains and really solve clinical problems.

Data availability statement

The original contributions presented in the study are included in the article/Supplementary Material, further inquiries can be directed to the corresponding authors.

Author contributions

DS and FS contributed to conception and design of the study. YW, AL, AI, MZ, LC, JS, LW, YZ, XZ, and ZX organized the database. JW, YX, and XW wrote the sections of the manuscript. All authors contributed to the article and approved the submitted version.

Funding

This work was supported in part by the National Key Research and Development Program of China (2022YFE0205700), National Natural Science Foundation of China (82227807), and National Key Technologies R&D Program of China (82027808).

Conflict of interest

JW, YX, XW, AL, YW, LC, JS, LW, YZ, XZ, ZX, FS, and DS are employees of Shanghai United Imaging Intelligence Co., Ltd, Shanghai, China. AI and MZ are employees of United Imaging Intelligence Co., Ltd., Cambridge, MA, United States. These companies have no role in designing and performing the surveillance and analyzing and interpreting the data.

Publisher's note

All claims expressed in this article are solely those of the authors and do not necessarily represent those of their affiliated organizations, or those of the publisher, the editors and the reviewers. Any product that may be evaluated in this article, or claim that may be made by its manufacturer, is not guaranteed or endorsed by the publisher.

Supplementary material

The Supplementary Material for this article can be found online at: https://www.frontiersin.org/articles/10.3389/fradi.2023.1153784/full#supplementary-material.

References

1. Aerts HJ. The potential of radiomic-based phenotyping in precision medicine: a review. JAMA Oncol. (2016) 2:1636–42. doi: 10.1001/jamaoncol.2016.2631

2. Galldiks N, Albert NL, Sommerauer M, Grosu AL, Ganswindt U, Law I, et al. PET Imaging in patients with meningioma-report of the RANO/PET group. Neuro Oncol. (2017) 19:1576–87. doi: 10.1093/neuonc/nox112

3. Kermany DS, Goldbaum M, Cai W, Valentim CCS, Liang H, Baxter SL, et al. Identifying medical diagnoses and treatable diseases by image-based deep learning. Cell. (2018) 172:1122–31. doi: 10.1016/j.cell.2018.02.010

4. Thoeny HC, Barbieri S, Froehlich JM, Turkbey B, Choyke PL. Functional and targeted lymph node imaging in prostate cancer: current status and future challenges. Radiology. (2017) 285:728–43. doi: 10.1148/radiol.2017161517

5. Torigian DA, Zaidi H, Kwee TC, Saboury B, Udupa JK, Cho ZH, et al. PET/MR imaging: technical aspects and potential clinical applications. Radiology. (2013) 267:26–44. doi: 10.1148/radiol.13121038/-/DC1

6. Fink JR, Muzi M, Peck M, Krohn KA. Multimodality brain tumor imaging: mR imaging, PET, and PET/MR imaging. J Nucl Med. (2015) 56:1554–61. doi: 10.2967/jnumed.113.131516

7. Fowler AM, Strigel RM. Clinical advances in PET-MRI for breast cancer. Lancet Oncol. (2022) 23:e32–43. doi: 10.1016/s1470-2045(21)00577-5

8. Zhang L, Wang Y, Peng Z, Weng Y, Fang Z, Xiao F, et al. The progress of multimodal imaging combination and subregion based radiomics research of cancers. Int J Biol Sci. (2022) 18:3458–69. doi: 10.7150/ijbs.71046

9. Minaee S, Boykov Y, Porikli F, Plaza A, Kehtarnavaz N, Terzopoulos D. Image segmentation using deep learning: a survey. IEEE Trans Pattern Anal Mach Intell. (2022) 44:3523–42. doi: 10.1109/TPAMI.2021.3059968

10. Moen E, Bannon D, Kudo T, Graf W, Covert M, Van Valen D. Deep learning for cellular image analysis. Nat Methods. (2019) 16:1233–46. doi: 10.1038/s41592-019-0403-1

11. Jin L, Shi F, Chun Q, Chen H, Ma Y, Wu S, et al. Artificial intelligence neuropathologist for glioma classification using deep learning on hematoxylin and eosin stained slide images and molecular markers. Neuro Oncol. (2021) 23:44–52. doi: 10.1093/neuonc/noaa163

12. Fuyong X, Yuanpu X, Hai S, Fujun L, Lin Y. Deep learning in microscopy image analysis: a survey. IEEE Trans Neural Netw Learn Syst. (2018) 29:4550–68. doi: 10.1109/TNNLS.2017.2766168

13. Funke J, Tschopp F, Grisaitis W, Sheridan A, Singh C, Saalfeld S, et al. Large scale image segmentation with structured loss based deep learning for connectome reconstruction. IEEE Trans Pattern Anal Mach Intell. (2019) 41:1669–80. doi: 10.1109/TPAMI.2018.2835450

14. Oksuz I, Clough JR, Ruijsink B, Anton EP, Bustin A, Cruz G, et al. Deep learning-based detection and correction of cardiac MR motion artefacts during reconstruction for high-quality segmentation. IEEE Trans Med Imaging. (2020) 39:4001–10. doi: 10.1109/TMI.2020.3008930

15. Gibson E, Giganti F, Hu Y, Bonmati E, Bandula S, Gurusamy K, et al. Automatic multi-organ segmentation on abdominal CT with dense V-networks. IEEE Trans Med Imaging. (2018) 37:1822–34. doi: 10.1109/tmi.2018.2806309

16. Isensee F, Jaeger PF, Kohl SAA, Petersen J, Maier-Hein KH. nnU-Net: a self-configuring method for deep learning-based biomedical image segmentation. Nat Methods. (2021) 18:203–11. doi: 10.1038/s41592-020-01008-z

17. de Vos BD, Berendsen FF, Viergever MA, Sokooti H, Staring M, Išgum I. A deep learning framework for unsupervised affine and deformable image registration. Med Image Anal. (2019) 52:128–43. doi: 10.1016/j.media.2018.11.010

18. Balakrishnan G, Zhao A, Sabuncu MR, Guttag J, Dalca AV. Voxelmorph: a learning framework for deformable medical image registration. IEEE Trans Med Imaging. (2019) 38:1788–800. doi: 10.1109/TMI.2019.2897538

19. Kang M, Hu X, Huang W, Scott MR, Reyes M. Dual-stream pyramid registration network. Med Image Anal. (2022) 78:102379. doi: 10.1016/j.media.2022.102379

20. Amor B B, Arguillere S, Shao L. ResNet-LDDMM: advancing the LDDMM framework using deep residual networks. IEEE Trans Pattern Anal Mach Intell. (2023) 45:3707–20. doi: 10.1109/TPAMI.2022.3174908

21. Xu J, Pan Y, Pan X, Hoi S, Yi Z, Xu Z. Regnet: self-regulated network for image classification. IEEE Trans Neural Netw Learn Syst. (2022) in press. doi: 10.1109/TNNLS.2022.3158966. [Epub ahead of print]

22. Wang H, Wang S, Qin Z, Zhang Y, Li R, Xia Y. Triple attention learning for classification of 14 thoracic diseases using chest radiography. Med Image Anal. (2021) 67:101846. doi: 10.1016/j.media.2020.101846

23. Hu JF, Huang TZ, Deng LJ, Jiang TX, Vivone G, Chanussot J. Hyperspectral image super-resolution via deep spatiospectral attention convolutional neural networks. IEEE Trans Neural Netw Learn Syst. (2021) 33:7251-65–00. doi: 10.1109/TNNLS.2021.3084682

24. Ji Z, Wang H, Han J, Pang Y. SMAN: stacked multimodal attention network for cross-modal image-text retrieval. IEEE Trans Cybern. (2022) 52:1086–97. doi: 10.1109/TCYB.2020.2985716

25. Tan H, Liu X, Yin B, Li X. DR-GAN: distribution regularization for text-to-image generation. IEEE Trans Neural Netw Learn Syst. (2022) in press. doi: 10.1109/TNNLS.2022.3165573. [Epub ahead of print]

26. Chikontwe P, Luna M, Kang M, Hong KS, Ahn JH, Park SH. Dual attention multiple instance learning with unsupervised complementary loss for COVID-19 screening. Med Image Anal. (2021) 72:102105. doi: 10.1016/j.media.2021.102105

27. Sobie EA. An introduction to MATLAB. Sci Signal. (2011) 4:tr7. doi: 10.1126/scisignal.2001984

28. Fedorov A, Beichel R, Kalpathy-Cramer J, Finet J, Fillion-Robin JC, Pujol S, et al. 3D Slicer as an image computing platform for the quantitative imaging network. Magn Reson Imaging. (2012) 30:1323–41. doi: 10.1016/j.mri.2012.05.001

29. Chan HY, Walker PS. Opensim as a preliminary kinematic testing platform for the development of total knee arthroplasty implants. J Biomech. (2018) 76:53–60. doi: 10.1016/j.jbiomech.2018.05.014

30. Song XW, Dong ZY, Long XY, Li SF, Zuo XN, Zhu CZ, et al. REST: a toolkit for resting-state functional magnetic resonance imaging data processing. PLoS One. (2011) 6:e25031. doi: 10.1371/journal.pone.0025031

31. Jiang H, van Zijl PC, Kim J, Pearlson GD, Mori S. Dtistudio: resource program for diffusion tensor computation and fiber bundle tracking. Comput Methods Programs Biomed. (2006) 81:106–16. doi: 10.1016/j.cmpb.2005.08.004

32. Wolf I, Vetter M, Wegner I, Bottger T, Nolden M, Schobinger M, et al. The medical imaging interaction toolkit. Med Image Anal. (2005) 9:594–604. doi: 10.1016/j.media.2005.04.005

33. Duan Q, Wang G, Wang R, Fu C, Li X, Wang N, et al. SenseCare: A research platform for medical image informatics and interactive 3D visualization. arXiv. (2022). doi: 10.48550/arXiv.2004.07031

34. Besson FL, Henry T, Meyer C, Chevance V, Roblot V, Blanchet E, et al. Rapid contour-based segmentation for $^{(18)}$F-FDG PET imaging of lung tumors by using ITK-SNAP: comparison to expert-based segmentation. Radiology. (2018) 288:277–84. doi: 10.1148/radiol.2018171756

35. Canny J. A computational approach to edge detection. IEEE Trans Pattern Anal Mach Intell. (1986) 8:679–98. doi: 10.1109/TPAMI.1986.4767851

36. The OpenCV Library. https://opencv.org/author/opencv/

37. LeCun Y, Bengio Y, Hinton G. Deep learning. Nature. (2015) 521:436–44. doi: 10.1038/nature14539

38. He K, Zhang X, Ren S, Sun J. Deep residual learning for image recognition. 2016 IEEE conference on computer vision and pattern recognition (CVPR) (2016). p. 770–8. doi: 10.48550/arXiv.1512.03385

39. Ronneberger O, Fischer P, Brox T. U-Net convolutional networks for biomedical segmentation. 2015 Medical image computing and computer-assisted intervention (MICCAI) (2015). p. 234–41. doi: 10.1007/978-3-319-24574-4_28

40. Wang X, Peng Y, Lu L, Lu Z, Bagheri M, Summers RM. Chestx-ray8: hospital-scale chest x-ray database and benchmarks on weakly-supervised classification and localization of common thorax diseases. 2017 IEEE conference on computer vision and pattern recognition (CVPR) (2017). p. 3462–71. doi: 10.1109/CVPR.2017.369

41. Shi F, Hu W, Wu J, Han M, Wang J, Zhang W, et al. Deep learning empowered volume delineation of whole-body organs-at-risk for accelerated radiotherapy. Nat Commun. (2022) 13:6566. doi: 10.1038/s41467-022-34257-x

42. Han M, Yao G, Zhang W, Mu G, Zhan Y, Zhou X, et al. Segmentation of CT thoracic organs by multi-resolution VB-nets. Proceedings of the 2019 challenge on segmentation of thoracic organs at risk in CT images (SegTHOR@ISBI 2019) (2019).

43. Milletari F, Navab N, Ahmadi S-A. V-Net: fully convolutional neural networks for volumetric medical image segmentation. 2016 Fourth international conference on 3D vision (3DV) (2016). p. 565–71. doi: 10.48550/arXiv.1606.04797

44. Linte CA, Fei B, Rong C, Zhou Q, Zhou XS, Zhan YJ, et al. Large-scale evaluation of V-net for organ segmentation in image guided radiation therapy. In Medical imaging 2019: image-guided procedures, robotic interventions, and modeling. (2019) Vol. 10951. p. 109510O1–10O7. doi: 10.1117/12.2512318

45. Hill DLG, Batchelor PG, Holden M, Hawkes DJ. Medical image registration. Phys Med Biol. (2001) 46:R1–45. doi: 10.1088/0031-9155/46/3/201

46. Hu Y, Modat M, Gibson E, Li W, Ghavami N, Bonmati E, et al. Weakly-supervised convolutional neural networks for multimodal image registration. Med Image Anal. (2018) 49:1–13. doi: 10.1016/j.media.2018.07.002

47. Zhu W, Myronenko A, Xu Z, Li W, Roth H, Huang Y, et al. NeurReg: neural registration and its application to image segmentation. In Proceedings of the IEEE/CVF winter conference on applications of computer vision (2020). p. 3617–26. doi: 10.1109/WACV45572.2020.9093506

48. Hara K, Kataoka H, Satoh Y. Can spatiotemporal 3D CNNs retrace the history of 2D CNNs and ImageNet? 2018 IEEE/CVF conference on computer vision and pattern recognition (2018). p. 6546–55. doi: 10.1109/CVPR.2018.00685

49. Zhou B, Khosla A, Lapedriza A, Oliva A, Torralba A. Learning deep features for discriminative localization. 2016 IEEE conference on computer vision and pattern recognition (CVPR) (2016). p. 2921–9. doi: 10.48550/arXiv.1512.04150

50. Selvaraju RR, Cogswell M, Das A, Vedantam R, Parikh D, Batra D. Grad-CAM: visual explanations from deep networks via gradient-based localization. 2017 IEEE international conference on computer vision (ICCV) (2017). p. 618–26. doi: 10.1109/ICCV.2017.74

51. Lin TY, Goyal P, Girshick R, He K, Dollar P. Focal loss for dense object detection. IEEE Trans Pattern Anal Mach Intell. (2020) 42:318–27. doi: 10.1109/TPAMI.2018.2858826

52. Chen K, Lin W, Li J, See J, Wang J, Zou J. AP-loss for accurate one-stage object detection. IEEE Trans Pattern Anal Mach Intell. (2021) 43:3782–98. doi: 10.1109/TPAMI.2020.2991457

53. Iandola F, Moskewicz M, Karayev S, Girshick R, Darrell T, Keutzer K. DenseNet: Implementing efficient ConvNet descriptor pyramids. arXiv. (2014). doi: 10.48550/arXiv.1404.1869

54. Tan M, Le QV. EfficientNet: Rethinking models scaling for convolutional neural networks. arXiv. (2019). doi: 10.48550/arXiv.1905.11946

55. Lambin P, Rios-Velazquez E, Leijenaar R, Carvalho S, van Stiphout RG, Granton P, et al. Radiomics: extracting more information from medical images using advanced feature analysis. Eur J Cancer. (2012) 48:441–6. doi: 10.1016/j.ejca.2011.11.036

56. Park SH, Lim H, Bae BK, Hahm MH, Chong GO, Jeong SY, et al. Robustness of magnetic resonance radiomic features to pixel size resampling and interpolation in patients with cervical cancer. Cancer Imaging. (2021) 21:19. doi: 10.1186/s40644-021-00388-5

57. Li Y, Lu L, Xiao M, Dercle L, Huang Y, Zhang Z, et al. CT Slice thickness and convolution kernel affect performance of a radiomic model for predicting EGFR status in non-small cell lung cancer: a preliminary study. Sci Rep. (2018) 8:17913. doi: 10.1038/s41598-018-36421-0

58. Whybra P, Parkinson C, Foley K, Staffurth J, Spezi E. Assessing radiomic feature robustness to interpolation in $^{(18)}$F-FDG PET imaging. Sci Rep. (2019) 9:9649. doi: 10.1038/s41598-019-46030-0

59. Park D, Oh D, Lee M, Lee SY, Shin KM, Jun JS, et al. Importance of CT image normalization in radiomics analysis: prediction of 3-year recurrence-free survival in non-small cell lung cancer. Eur Radiol. (2022) 32:8716–25. doi: 10.1007/s00330-022-08869-2

60. Zwanenburg A, Vallieres M, Abdalah MA, Aerts H, Andrearczyk V, Apte A, et al. The image biomarker standardization initiative: standardized quantitative radiomics for high-throughput image-based phenotyping. Radiology. (2020) 295:328–38. doi: 10.1148/radiol.2020191145

61. Patro SGK, sahu KK. Normalization: A preprocessing stage. arXiv. (2015). doi: 10.48550/arXiv.1503.06462

62. Hampel H, Cummings J, Blennow K, Gao P, Jack CR Jr., Vergallo A. Developing the ATX(N) classification for use across the Alzheimer disease continuum. Nat Rev Neurol. (2021) 17:580–9. doi: 10.1038/s41582-021-00520-w

63. Myszczynska MA, Ojamies PN, Lacoste AMB, Neil D, Saffari A, Mead R, et al. Applications of machine learning to diagnosis and treatment of neurodegenerative diseases. Nat Rev Neurol. (2020) 16:440–56. doi: 10.1038/s41582-020-0377-8

64. Liu S, Jie C, Zheng W, Cui J, Wang Z. Investigation of underlying association between whole brain regions and Alzheimer's Disease: a research based on an artificial intelligence model. Front Aging Neurosci. (2022) 14:872530. doi: 10.3389/fnagi.2022.872530

65. Zuo XN, Anderson JS, Bellec P, Birn RM, Biswal BB, Blautzik J, et al. An open science resource for establishing reliability and reproducibility in functional connectomics. Sci Data. (2014) 1:140049. doi: 10.1038/sdata.2014.49

66. Gu D, Shi F, Hua R, Wei Y, Li Y, Zhu J, et al. An artificial-intelligence-based age-specific template construction framework for brain structural analysis using magnetic resonance images. Hum Brain Mapp. (2022) 44:861–75. doi: 10.1002/hbm.26126

67. Korf ESC, Wahlund LO, Visser PJ, Scheltens P. Medial temporal lobe atrophy on MRI predicts dementia in patients with mild cognitive impairment. Neurology. (2004) 63:94–100. doi: 10.1212/01.wnl.0000133114.92694.93

68. Liu T, Siegel E, Shen D. Deep learning and medical image analysis for COVID-19 diagnosis and prediction. Annu Rev Biomed Eng. (2022) 24:179–201. doi: 10.1146/annurev-bioeng-110220-012203

69. Minaee S, Kafieh R, Sonka M, Yazdani S, Jamalipour Soufi G. Deep-COVID: predicting COVID-19 from chest x-ray images using deep transfer learning. Med Image Anal. (2020) 65:101794. doi: 10.1016/j.media.2020.101794

70. Wang G, Liu X, Shen J, Wang C, Li Z, Ye L, et al. A deep-learning pipeline for the diagnosis and discrimination of viral, non-viral and COVID-19 pneumonia from chest x-ray images. Nat Biomed Eng. (2021) 5:509–21. doi: 10.1038/s41551-021-00704-1

71. Wang S, Zha Y, Li W, Wu Q, Li X, Niu M, et al. A fully automatic deep learning system for COVID-19 diagnostic and prognostic analysis. Eur Respir J. (2020) 56:2000775. doi: 10.1183/13993003.00775-2020

72. Goncharov M, Pisov M, Shevtsov A, Shirokikh B, Kurmukov A, Blokhin I, et al. CT-based COVID-19 triage: deep multitask learning improves joint identification and severity quantification. Med Image Anal. (2021) 71:102054. doi: 10.1016/j.media.2021.102054

73. Wang Y, Chen Y, Wei Y, Li M, Zhang Y, Zhang N, et al. Quantitative analysis of chest CT imaging findings with the risk of ARDS in COVID-19 patients: a preliminary study. Ann Transl Med. (2020) 8:594. doi: 10.21037/atm-20-3554

74. Zhu X, Song B, Shi F, Chen Y, Hu R, Gan J, et al. Joint prediction and time estimation of COVID-19 developing severe symptoms using chest CT scan. Med Image Anal. (2021) 67:101824. doi: 10.1016/j.media.2020.101824

75. Shi F, Xia L, Shan F, Song B, Wu D, Wei Y, et al. Large-scale screening of COVID-19 from community acquired pneumonia using infection size-aware classification. Phys Med Biol. (2021) 66:065031. doi: 10.1088/1361-6560/abe838

76. Ouyang X, Huo J, Xia L, Shan F, Liu J, Mo Z, et al. Dual-sampling attention network for diagnosis of COVID-19 from community acquired pneumonia. IEEE Trans Med Imaging. (2020) 39:2595–605. doi: 10.1109/TMI.2020.2995508

77. Roos EM, Arden NK. Strategies for the prevention of knee osteoarthritis. Nat Rev Rheumatol. (2016) 12:92–101. doi: 10.1038/nrrheum.2015.135

78. Chaudhari AS, Kogan F, Pedoia V, Majumdar S, Gold GE, Hargreaves BA. Rapid knee MRI acquisition and analysis techniques for imaging osteoarthritis. J Magn Reson Imaging. (2020) 52:1321–39. doi: 10.1002/jmri.26991

79. Tang X, Guo D, Liu A, Wu D, Liu J, Xu N, et al. Fully automatic knee joint segmentation and quantitative analysis for osteoarthritis from magnetic resonance (MR) images using a deep learning model. Med Sci Monit. (2022) 28:e936733. doi: 10.12659/MSM.936733

80. Shan F, Gao Y, Wang J, Shi W, Shi N, Han M, et al. Abnormal lung quantification in chest CT images of COVID-19 patients with deep learning and its application to severity prediction. Med Phys. (2021) 48:1633–45. doi: 10.1002/mp.14609

81. Joensuu H, Hohenberger P, Corless CL. Gastrointestinal stromal tumour. Lancet. (2013) 382:973–83. doi: 10.1016/s0140-6736(13)60106-3

82. Nilsson B, Bumming P, Meis-Kindblom JM, Oden A, Dortok A, Gustavsson B, et al. Gastrointestinal stromal tumors: the incidence, prevalence, clinical course, and prognostication in the preimatinib mesylate era–A population-based study in western Sweden. Cancer. (2005) 103:821–9. doi: 10.1002/cncr.20862

83. Kang B, Yuan X, Wang H, Qin S, Song X, Yu X, et al. Preoperative CT-based deep learning model for predicting risk stratification in patients with gastrointestinal stromal tumors. Front Oncol. (2021) 11:750875. doi: 10.3389/fonc.2021.750875

84. Qian L, Zhou Q, Cao X, Shen W, Suo S, Ma S, et al. A cascade-network framework for integrated registration of liver DCE-MR images. Comput Med Imaging Graph. (2021) 89:101887. doi: 10.1016/j.compmedimag.2021.101887

85. Si N, Shi K, Li N, Dong X, Zhu C, Guo Y, et al. Identification of patients with acute myocardial infarction based on coronary CT angiography: the value of pericoronary adipose tissue radiomics. Eur Radiol. (2022) 32:6868–77. doi: 10.1007/s00330-022-08812-5

86. Huang X, Shu J, Yan Y, Chen X, Yang C, Zhou T, et al. Feasibility of magnetic resonance imaging-based radiomics features for preoperative prediction of extrahepatic cholangiocarcinoma stage. Eur J Cancer. (2021) 155:227–35. doi: 10.1016/j.ejca.2021.06.053

87. Qian X, Lu X, Ma X, Zhang Y, Zhou C, Wang F, et al. A multi-parametric radiomics nomogram for preoperative prediction of microvascular invasion status in intrahepatic cholangiocarcinoma. Front Oncol. (2022) 12:838701. doi: 10.3389/fonc.2022.838701

88. Wang S, Zhao Z, Ouyang X, Wang Q, Shen D. ChatCAD: Interactive computer-aided diagnosis on medical image using large language models. arXiv. (2023). doi: 10.48550/arXiv.2302.07257
